# Characteristics, changes and influence of body composition during a 4486 km transcontinental ultramarathon: results from the Transeurope Footrace mobile whole body MRI-project

**DOI:** 10.1186/1741-7015-11-122

**Published:** 2013-05-08

**Authors:** Uwe HW Schütz, Christian Billich, Kathrin König, Christian Würslin, Heike Wiedelbach, Hans-Jürgen Brambs, Jürgen Machann

**Affiliations:** 1 Department of Diagnostic and Interventional Radiology, University Hospital of Ulm, Albert-Einstein-Allee 23, 89081, Ulm, Germany; 2 Outpatient Rehabilitation Centre at University Hospital of Ulm, Pfarrer-Weiß-Weg 10, 89073, Ulm, Germany; 3 Section on Experimental Radiology, Department of Diagnostic and Interventional Radiology, University Hospital of Tübingen, Tübingen, Germany; 4 Institute for Diabetes Research and Metabolic Diseases (IDM), Metabolic Imaging – of the Helmholtz Center Munich at University of Tübingen (Paul Langerhaus Institute Tübingen), Tübingen, Germany

**Keywords:** Magnetic resonance imaging, MRI, Body mass, Body volume, Body composition, Running, Marathon, Ultramarathon, Performance, Adipose tissue, Body fat, Lean tissue, Visceral, Somatic, Topography, Segmentation, Mapping

## Abstract

**Background:**

Almost nothing is known about the medical aspects of runners doing a transcontinental ultramarathon over several weeks. The results of differentiated measurements of changes in body composition during the Transeurope Footrace 2009 using a mobile whole body magnetic resonance (MR) imager are presented and the proposed influence of visceral and somatic adipose and lean tissue distribution on performance tested.

**Methods:**

22 participants were randomly selected for the repeated MR measurements (intervals: 800 km) with a 1.5 Tesla MR scanner mounted on a mobile unit during the 64-stage 4,486 km ultramarathon. A standardized and validated MRI protocol was used: T1 weighted turbo spin echo sequence, echo time 12 ms, repetition time 490 ms, slice thickness 10 mm, slice distance 10 mm (breath holding examinations). For topographic tissue segmentation and mapping a modified fuzzy c-means algorithm was used. A semi-automatic post-processing of whole body MRI data sets allows reliable analysis of the following body tissue compartments: Total body volume (TV), total somatic (TSV) and total visceral volume (TVV), total adipose (TAT) and total lean tissue (TLT), somatic (SLT) and visceral lean tissue (VLT), somatic (SAT) and visceral adipose tissue (VAT) and somatic adipose soft tissue (SAST). Specific volume changes were tested on significance. Tests on difference and relationship regarding prerace and race performance and non-finishing were done using statistical software SPSS.

**Results:**

Total, somatic and visceral volumes showed a significant decrease throughout the race. Adipose tissue showed a significant decrease compared to the start at all measurement times for TAT, SAST and VAT. Lean adipose tissues decreased until the end of the race, but not significantly. The mean relative volume changes of the different tissue compartments at the last measurement compared to the start were: TV −9.5% (SE 1.5%), TSV −9.4% (SE 1.5%), TVV −10.0% (SE 1.4%), TAT −41.3% (SE 2.3%), SAST −48.7% (SE 2.8%), VAT −64.5% (SE 4.6%), intraabdominal adipose tissue (IAAT) −67.3% (SE 4.3%), mediastinal adopose tissue (MAT) −41.5% (SE 7.1%), TLT −1.2% (SE 1.0%), SLT −1.4% (SE 1.1%). Before the start and during the early phase of the Transeurope Footrace 2009, the non-finisher group had a significantly higher percentage volume of TVV, TAT, SAST and VAT compared to the finisher group. VAT correlates significantly with prerace training volume and intensity one year before the race and with 50 km- and 24 hour-race records. Neither prerace body composition nor specific tissue compartment volume changes showed a significant relationship to performance in the last two thirds of the Transeurope Footrace 2009.

**Conclusions:**

With this mobile MRI field study the complex changes in body composition during a multistage ultramarathon could be demonstrated in detail in a new and differentiated way. Participants lost more than half of their adipose tissue. Even lean tissue volume (mainly skeletal muscle tissue) decreased due to the unpreventable chronic negative energy balance during the race. VAT has the fastest and highest decrease compared to SAST and lean tissue compartments during the race. It seems to be the most sensitive morphometric parameter regarding the risk of non-finishing a transcontinental footrace and shows a direct relationship to prerace-performance. However, body volume or body mass and, therefore, fat volume has no correlation with total race performances of ultra-athletes finishing a 4,500 km multistage race.

## Background

With the worldwide growing number of people running, endurance sports have experienced differentiation into multiple (sub-) disciplines in the last decades. Concerning distance running, the ultramarathon (UM) seems to be the greatest challenge in endurance running. The German Ultramarathon Association (DUV) defines footraces of 50 km or longer as UM. However, as in every field of human physical activities, some people try to push themselves to the limits and beyond. For these ultra-athletes a multistage ultramarathon (MSUM) is the ultimate test of endurance performance. Sometimes, the worldwide small group of ultra-endurance runners meet with each other trying to achieve the impossible: finishing a multistage transcontinental footrace over thousands of kilometers. These most extreme multistage endurance competitions in the world take the runner to a different level, where nutrition, sleep, energy and psychological states have to be carefully managed. Besides a few case reports, almost nothing has been reported about the medical aspects of runners doing a transcontinental extended MSUM over several weeks [1]. Until now, there have been no series published regarding UM running over more than 1,500 km. However, prolonged ultra-endurance footraces offer the best opportunity to study physical adaptations and the relationships of the physiological parameters in endurance athletes.

The Transeurope Footrace Project (TEFR-project) [2] is the first observational cohort field study of a transcontinental MSUM, the Transeurope Footrace 2009 (TEFR09) [3]. A unique group of 67 endurance runners (mean age 50.7 years, standard deviation (SD) 10.5 years, range 26 to 74 years, m 56 (83.6%)) met the challenge and tried to cross six countries while running 4,486 km in 64 stages (mean 70.1 km, min 44 km, max 95.1 km) without any day of rest [4]. The central aspect of the TEFR-project was the use of a mobile magnetic resonance imaging (MRI) scanner accompanying the TEFR09 participants on a truck trailer over 64 days under their ‘natural’ conditions [2].

One focus of this presentation is on the descriptive presentation of characteristics and changes in body composition during TEFR09 in a new way, differentiating between somatic and visceral and segmental volumes of defined fat and lean tissue compartments measured by continuous mobile whole body MRI. In addition, possible associations of body volume composition and prerace and race performance were analyzed to test the following hypotheses: it is hypothesized, that prerace endurance running performance is related to specific body fat and lean tissue composition in ultra-athletes. Secondarily, it is supposed, that although the running distance of a transcontinental UM cannot be trained for concerning the running volume (km), participants need specific prerace performance skills and fat and lean tissue volume distribution, if they want to finish such a race. Due to the expected huge energy burden a transcontinental footrace without any day of rest implies, another assumption is that it is mandatory for every participant to lose body mass and total body volume (TV) due to massive adipose tissue decrease and more or less lean tissue catabolism. At least, with the continuous differentiated measurement of body tissue compartments throughout the entire TEFR09, it should be shown indirectly, that although the participants are preselected in regard to their ultra endurance running expertise, they will develop further economical adaptations as the 4,500 km race progresses.

## Methods

### Subjects

Every TEFR09 participant was asked to join the TEFR-project, which was approved by the local ethics committee of the University Hospital of Ulm (UHU, No.: 270/08-UBB/se) in accordance with the Declaration of Helsinki, regarding the study design, risk management plan and individual protocols [2]. Forty-four participants (67%) were recruited for the study and gave their informed written consent. Every second subject (n = 22, 20 men, mean age 49.1 years, SD 11.5 yrs., range 27 to 69 years) was randomly selected for whole body MRI measurements regarding body composition. According to project protocol these subjects underwent a whole body MRI before the start at Bari (South Italy) and during the race in measurement intervals of approximately 800 km. Due to various reasons, deviations from planned measurement intervals (MI: t0 to t6) occurred. The mean deviation of actual from planned measurement intervals was 187.8 km (SD = 141.3 km) [2].

### Prerace performance

Before the start of TEFR09 all subjects filled out specific questionnaires concerning their prerace experience in endurance running. This history includes the years of regular endurance running (PRY), the number of finished (n_F_) marathons (M), UM and MSUM, and the prerace records (PRR) for marathon and specific UM (50 km, 100 km, 6 hours, 12 hours, 24 hours) races within the last decade before TEFR. It also includes the extent of prerace training (PRT) 16 months before TEFR09: training volume (Vol: km/week), training duration (Time: hours/week) and the training intensity (Int: km/hour). The self-disclosures about n_F_ and PRR were cross checked with the archive of the DUV and discrepancies were clarified. However, for PRT and PRY we had to rely solely on the self-disclosures; these could not be compared with any official lists.

### Body composition analysis

Different techniques for quantification of body fat are described and more or less commonly used in the literature:. *In vivo*, two-compartment model methods are the hydrodensitometry [5] and the body fat percentage and muscle mass calculation from anthropometric data such as skinfold thickness (SF) calipometry and/or segmental body circumferences (CF) [6-8]. Three-compartment methods are the bioelectrical impedance analysis (BIA) [9] and dual-energy X-ray absorptiometry (DEXA) [10]. Using these methods, indirect measurement, approximated calculation or simple estimation of total, regional or local adipose or lean tissue [11-14] is possible. In contrast, a whole body MRI assessment of adipose tissue as a multi-compartment method is the only method enabling exact topographic tissue mapping and tissue segmentation. Therefore, it is the gold standard imaging tool for differentiated assessment of adipose or lean tissue distribution in the body [15-18].

Subjects who finished TEFR09 had whole body MRI six times during TEFR09 (seven measurements in total). Measurement of body mass (BM) was done at the same time as MRI and every fourth day: BIA balance Tanita BC-545 to the nearest 0.1 kg (Tanita, Arlington Heights, IL, U.S.A.). Body height was measured with a wall-mounted stadiometer (to the nearest 5 mm, standing barefoot) and body mass index (BMI) was calculated.

### Mobile whole body MRI

For whole body magnetic resonance (MR) measurements a 1.5 Tesla MRI scanner (Magnetom Avantot™, Siemens Ltd., Erlangen, Germany) mounted on a mobile unit (MRI-Trailer, SMIT Mobile Equipment B.V., Great Britain) was used. The total 45 tonnes of equipment (MRI-trailer, truck tractor, external 105KVA diesel-generator, and material van) was built up and taken down daily at each stopover of TEFR09, requiring daily checks and support of all technical systems [2].

Several MRI techniques have been described for the measurement and quantification of body fat composition: T1-weighted imaging by spin-echo or gradient-echo techniques [14,16,19], chemical shift selective (CHESS) imaging [20-22], or DIXON techniques [23,24]. All of them have specific advantages and disadvantages, the details of which are beyond the scope of this article. For analysis of body composition a standardized assessment of whole body adipose tissue measurement based on a MRI protocol according to Machann *et al*. [25] was used. A two-dimensional T1-weighted turbo spin echo sequence with an echo train length of seven was applied (Siemens Ltd.). Measurement parameters were set to be: flip angle 180°, echo time 12 ms, repetition time 490 ms, slice thickness 10 mm, slice distance 10 mm, 5 slices per sequence, field of view 1,991 cm^2^, matrix size 256 × 196 was recorded in a measuring time of 12 seconds (allowing breath holding examinations in the trunk area), bandwidth 120 Hz/pixel. A total of 90 to 120 images were generated, depending on the size of the subject. Total examination time was between 20 and 25 minutes including one rearrangement of prone positioned subject (head forward and arms extended for upper body, feet forward for lower body), as total table feed of the MR-imager is limited to 110 cm. In order to guarantee identical slice positions after repositioning, the subjects were marked at the iliac crest. A body coil was used.

### Image post-processing

For topographic tissue segmentation and mapping of the athletes body a fuzzy c-means algorithm according to Würslin *et al*. [26] was used. This approach provides a simple and time-saving strategy for assessment and standardization of individual adipose tissue distribution in the entire body. Due to standardization using defined internal markers, it enables a completely automatic, reliable analysis and creation of adipose tissue distribution profiles of the whole body from the multislice MR datasets and makes a reliable comparison of subjects with different body structure possible [25,26].

The signal of intestinal content with a short T1can be interpreted as visceral adipose tissue (VAT) in the absence of intraluminal gastroenteric nutrition fat (INF). If the scanned subject is in a non-fasting condition, the visceral T1 signal occurs from both, VAT and INF. Reliability (mean absolute deviation of three repeated measurements) is noted with 3.08% for total volume (TV), 1.48% for total adipose tissue (TAT) and 1.13% for visceral adipose tissue (VAT) [26].

**Figure 1:**
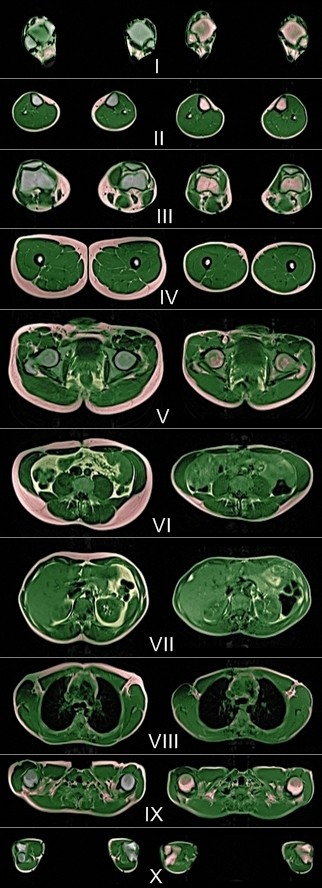
**Semiautomatic separation of adipose bone marrow**: **selected slices from whole body MRI of a 32-year-old male finisher of TEFR09.** I: ankles, II: middle of lower legs, III: knees, IV: middle of upper legs, V: hip/pelvis, VI: umbilical level, VII: upper abdomen, VIII: heart/mediastinum, IX: shoulder girth, X: elbows. Left row: before start (t0), green: TLT, red: SAST, yellow: VAT+INF, blue: ABM. Right row: after 4,120 km of running (t5), green: TLT, red: SAT (=SAST + ABM), yellow: VAT + INF. ABM, adipose bone marrow; INF, intraluminal nutrition fat; MRI, magnetic resonance imaging; SAST, somatic adipose soft tissue; SAT, somatic adipose tissue; TEFR09, Transeurope Footrace 2009; TLT, total lean tissue; VAT, visceral adipose tissue.

**Figure 2:**
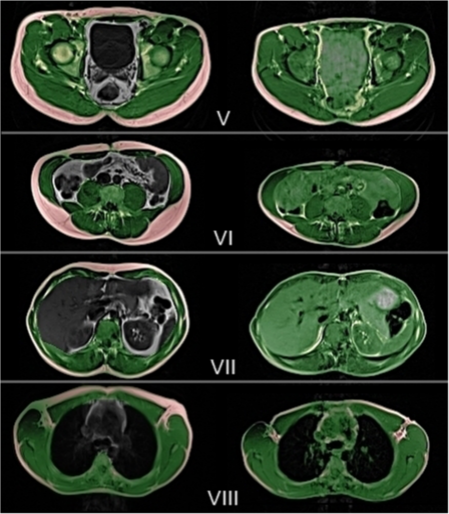
**Semiautomatic separation of somatic and visceral volume (right row) and intraluminal nutrition fat (left row): selected slices from whole body MRI of a 32-year-old male finisher of TEFR09.** V: hip/pelvis, VI: umbilical level, VII: upper abdomen, VIII: heart/mediastinum. Left row: before start (t0), green: SLT, red: TSAT, grey: TVV. Right row: after 4,120 km of running (t5), green: TLT, red: SAT (=SAST+ABM), yellow: VAT, blue: INF. ABM, adipose bone marrow; INF, intraluminal nutrition fat; MRI, magnetic resonance imaging; SAT, somatic adipose tissue; SLT, somatic lean tissue; TEFR09, Transeurope Footrace 2009; TLT, total lean tissue; TSAT, total somatic adipose tissue; TVV, total visceral volume; VAT, visceral adipose tissue.

Due to their immense mental and physical stress caused by the daily ultra-endurance burden, the runners’ biggest fear was losing too much energy over the course of TEFR09. Their primary effort after stage finishing was to get as much nutrition and calories as possible before falling asleep. Therefore, it was not always possible to ensure fasting conditions of the subjects for mobile MRI measurements. Some subjects were motivated enough to do the MR examination directly after the daily stage before eating in a fasting but exhausted condition, so they sometimes were not able to lie absolutely still on the MR table and follow the breath commands exactly. These specific circumstances resulted in the image post-processing analysis being less automated than that mentioned by Machann and Würslin [25,26]: Movement artifacts had to be cleared manually more often before automatic post-processing. Compared to normal or overweight patients, in thin and lean bodies the amount of adipose bone marrow (ABM) and INF is more relevant in relation to whole body adipose and lean tissue. At the start of TEFR09, ABM and INF together accounted for 13.2% of total adipose tissue. Due to the continuous loss of adipose body tissue, this ratio rises up to 28.2% until the end of the race. For visceral adipose tissue, INF rose from 3% at the start up to 65.4% at the end of TEFR09. Therefore, a manual separation of ABM (Figure 1) and INF (Figure 2) was done on all MR slices of the subjects. Looking at the mean differences, Würslin *et al*. [26] calculated between manual tissue segmentation and their automatic procedure (2.07% for TV, 8.13% for TAT, 3.21% for VAT), the described additional manual corrections regarding the small volumes of ABM and INF are appropriate.

After these procedures a specific and extensive topographic mapping and segmentation of body tissue was possible (Table 1). Total volume (TV) can be subdivided into total somatic volume (TSV) and total visceral volume (TVV, Figure 2) or can be subdivided into total adipose tissue (TAT; without INF) and total lean tissue (TLT). TLT can be separated into somatic (SLT) and visceral lean tissue (VLT). Subtraction of ABM from TAT leads to total adipose soft tissue (TAST). TAST can be subdivided into VAT and somatic adipose soft tissue (SAST). Therefore, somatic adipose tissue (SAT, Figure 1) is the same as SAST plus ABM or TAT minus VAT, respectively. VAT can be subdivided into intraabdominal (retro- and intraperitoneal) adipose tissue (IAAT) and intrathoracic, mainly mediastinal adipose tissue (MAT). Body segmentation was done into upper extremities (UE), trunk (TR) and lower extremities (LE). TV, lean tissue (LT) and SAST volume was calculated for the upper and lower extremities (UE and LE) and for the trunk (TR). For nomenclature of specific segmented tissues see Table 1.

**Table 1:** Abbreviations of compartments after tissue mapping and segmentation with mobile whole body MRI data sets (T2*)

**Abbreviation**	**Description, Definition**
	**Tissue mapping in specific compartments**
ABM	Adipose bone marrow
TV	Total volume of the body (from ankle to wrist), without INF
TVV	Total visceral volume: includes intrathoracic and intraabdominal volume.
TSV	Total somatic volume (TV without TVV).
TLT	Total lean tissue
VLT	Visceral lean tissue: includes lean tissue of intrathoracic and intraabdominal organs.
SLT	Somatic lean tissue: mostly muscles
TAT	Total adipose tissue (without INF)
SAT	Somatic adipose tissue (TAT without VAT)
TAST	Total adipose soft tissue (TAT without ABM)
SAST	Somatic adipose soft tissue (TAT without ABM and VAT)
SCAT	Subcutaneous adipose tissue (SAST without IMAT)
IMAT	Intermuscular adipose tissue (SAST without SCAT)
VAT	Visceral adipose tissue (IAAT + MAT without INF)
IAAT	Intraabdominal adipose tissue: retroperitoneal and intraperitoneal (mesenteric, omental) fat depots (without INF)
MAT	Intrathoracic, mainly mediastinal adipose tissue
INF	(Undigested) intraluminal nutrition fat in the gastrointestinal tract
	** Tissue mapping of body segments**
TV-LE	Total volume of lower extremities (apex trochanter major to ankle joint)
TV-TR	Total volume of trunk (acromion to apex of trochanter major)
TV-UE	Total volume of upper extremities (wrist to acromion level)
LT-LE	Lean tissue volume of lower extremities (apex trochanter major to ankle joint)
LT-TR	Lean soft tissue volume of trunk (acromion to apex of trochanter major)
LT-UE	Lean soft tissue volume of upper extremities (wrist to acromion level)
SAST-LE	Adipose soft tissue volume of lower extremities (apex trochanter major to ankle joint)
SAST-TR	Adipose soft tissue volume of trunk (acromion to apex of trochanter major)
SAST-UE	Adipose soft tissue volume of upper extremities (wrist to acromion level)

### Statistical analysis

For data elaboration specific software was used: Microsoft™ Office Excel™ (Release 12.0.6665.5003, Microsoft Home and Student Suite, 2007, Microsoft Inc.) for data documentation, SPSS (IBM™ SPSS™ Statistics, Release 19.0.0, 2010, SPSS Inc.) for statistical analysis and SigmaPlot for Windows Version 11.0 (Release 11.2.0.5, 2008, Systat Software Inc.,) for graphical data presentation.

The measured volumes of tissue compartments are presented as percentage volumes (vol%) and as absolute [1] and relative differences (%) to start. For every measurement interval (t0 to t5) the dispersion measures are presented graphically in box plot figures (median, 25th/75th percentile, 10th/90th percentile and all outliers) for all subjects (finishers and non-finishers) and location measures (mean and standard error, SE) are presented graphically in line figures for finishers only. Calculated total changes (t5 versus t0) of volumes and volume percentages are presented in text as means and standard deviation (SD) with minimum (min) and maximum (max) as appropriate.

### Analyses on volume changes during TEFR09

For analysis of the significance regarding volume changes of the specific tissue compartments during TEFR09, a univariate variance analysis (ANOVA) for repeated measurements was preferred (only subjects who had the whole body MRI at every measurement interval (t0 to t5): n = 12). Therefore, a common linear model for repeated measurements (with *post hoc* analysis on the significance between the different times of measurement) was chosen. For correction of accumulation of the alpha level due to multiple testing (of hypothesis: ‘The means at stage intervals are significantly different to means at start’), the Bonferroni-procedure for confidence-interval (CI) adaption was applied. For the univariate ANOVA model, one precondition, the sphericity of data (homogenity between the variance of differences of two measurements) is necessary and was proven by the Mauchly-Test. Because of the small number of subjects, the power of the Mauchly-Test regarding sphericity is low. Therefore, the ‘Greenhouse-Geisser’ (SPSS) correction procedure was used. Looking at result reliability and test power, in cases of severe injury of the sphericity assumption, a multivariate ANOVA test was used. In cases of missing values, the specific dependent variable (specific tissue compartment) was excluded from ANOVA analysis.

### Analyses of difference

For dependence analysis including all stages of and total TEFR09, analyses of difference between the dichotomous nominally scaled dependent variables of the sample finishing status (finisher/non-finisher: F/NF) regarding prerace running performance history and regarding total, lean and adipose tissue volumes were carried out. Depending on normal or free distribution of the independent interval scaled variables, the parametric independent t-test (variance homogeneity was calculated with Levene`s test) or nonparametric Mann–Whitney-U-test was used. Due to the higher power in small cohorts, the Shapiro-Wilk [27,28] test (and not the Kolmogorov-Smirnov statistic [29]) was used to check for normal distribution of the independent prerace variables of performance (PRY, PRT, PRR).

### Analysis of relationships

For analyses of relationships, the Pearson correlation coefficient (CC_P_) and Spearman-rho correlation coefficient (CC_S_) were calculated for parametric and non-parametric parameters, respectively, using bivariate (two-sided) or univariate (one-sided) testing as appropriate:

BM versus TV and its distribution throughout the race: bivariate CC_S_

Prerace performance versus percentage total, lean and adipose volumes: univariate CC_S_ / CC_P_

Race performance versus percentage total, lean and adipose volume_s_: univariate CC_P_

For interpretation of CC-values the effect size according to Cohen (r = 1: low, r = 3: medium, r = 5: high) was used [30]. For all tests, an alpha level (*P*-value) of 0.05 was used to indicate significance.

## Results

### Case presentation

**Figure 3:**
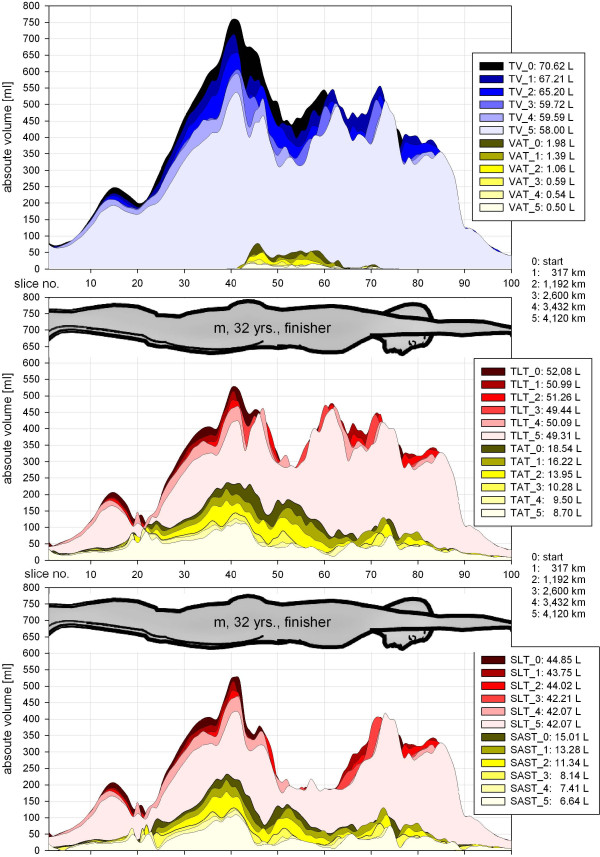
Topography of lean and adipose tissue changes in a 32-year-old male finisher.

Figure 3 shows the topographical mapping of changes of lean and adipose tissue of a subject (male, 32 years, finisher) with one of the largest decreases of SAST and VAT during TEFR09. Runners often had discomfort or pain after stage finishing, so the investigators tried to adapt body positioning in the MR scanner to the athletes’ current problems to make it as comfortable as possible for them. Therefore, a reliable and strictly standardized lying position on the MRI table was not possible at each time of measurement. Sometimes, knees or elbows were positioned more or less straightened. This explains the sometimes visible but small topographical phase shifting between different times of measurement in Figure 3.

### Total body volume versus body mass/body mass index

**Figure 4:**
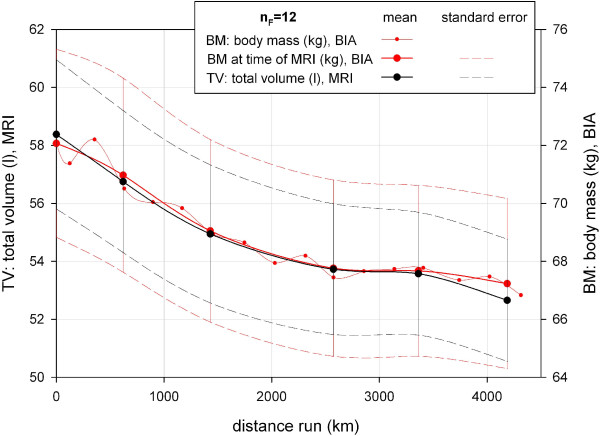
**Comparison of total body volume versus body mass during TEFR09 (finisher, n_F _= 12).** TEFR09, Transeurope Footrace 2009.

The absolute volumes of all investigated body tissue compartments and segments are shown in Table 2. Over all subjects, mean loss of BM and BMI at the end of the race was 5.23 kg (SD 3.72 kg) and 1.49 kg/m^2^ (SD 1.18 kg/m^2^), respectively (Tables 3 and 4). There was a high correlation (CC_S_: 0.978, *P* <0.001) between BM (kg) and TV [1] regarding mean absolute value changes throughout the race (Figure 4).

**Table 2:** Mean volumes (l) of body compartments and segments (all subjects)

**Abbreviation**	**t0: start**	**t1: 317 to 789 km**	**t2: 1,003 to 1,635 km**	**t3: 2,516 to 2,738 km**	**t4: 3,234 to 3,669 km**	**t5: 4,037 to 4,440 km**
**Tissue mapping in specific compartments**						
**TV**	57.70	56.63	54.75	53.39	54.16	52.65
**TVV**	8.74	8.34	7.93	7.64	7.65	7.58
**TSV**	48.97	48.29	46.82	45.74	46.51	45.08
**TLT**	44.34	44.61	45.07	45.38	46.20	45.52
**VLT**	7.02	7.04	7.01	7.10	7.14	7.12
**SLT**	37.32	37.56	38.06	38.28	39.06	38.40
**TAT**	13.36	12.02	9.68	8.00	7.96	7.14
**SAT**	11.65	10.73	8.76	7.46	7.45	6.68
**TAST**	11.52	10.19	7.76	6.05	6.01	5.19
**SAST**	9.81	8.89	6.84	5.51	5.50	4.73
**VAT**	1.71	1.30	0.92	0.54	0.51	0.45
**IAAT**	1.55	1.15	0.77	0.39	0.36	0.33
**MAT**	0.17	0.15	0.15	0.15	0.14	0.13
**INF**	0.05	0.21	0.36	0.57	0.70	0.86
**ABM**	1.98	1.93	1.92	1.95	1.95	1.95
**Tissue mapping of body segments**						
**TV-LE**	23.51	22.49	21.46	21.65	22.02	20.60
**TV-TR**	28.83	28.63	27.86	26.57	27.03	27.00
**TV-UE**	5.79	5.89	5.75	5.73	5.64	5.60
**TLT-LE**	17.51	16.97	16.82	17.45	17.83	16.97
**TLT-TR**	23.22	23.12	23.46	23.02	23.39	23.39
**TLT-UE**	4.79	4.62	4.70	4.81	4.74	4.77
**SAST-LE**	4.84	4.36	3.43	2.96	2.96	2.40
**SAST-TR**	4.31	3.47	2.12	1.02	0.99	0.81
**SAST-UE**	0.98	0.95	0.72	0.57	0.54	0.48

ABM, adipose bone marrow; IAAT, intraabdominal adipose tissue; INF, intraluminal nutrition fat; LE, lower extremities; MAT, mediastinal adipose tissue; SAST, somatic adipose soft tissue; SAT, somatic adipose tissue; SLT, somatic lean tissue; TAT, total adipose tissue; TLT, total lean tissue; TR, trunk; TSV, total somatic volume; TV, total volume; TVV, total visceral volume; UE, upper extremities; VAT, visceral adipose tissue; VLT, visceral lean tissue.

**Table 3:** BM and BMI loss during TEFR09

**Number**			**Distance run (km)**	**BM (kg)**						**BMI (kg/m**^**2**^**)**					
**all**	**F**	**NF**		**all**		**F**		**NF**		**all**		**F**		**NF**	
			**mean**	**mean**	**SD**	**mean**	**SD**	**mean**	**SD**	**mean**	**SD**	**mean**	**SD**	**mean**	**SD**
22	12	10	0.00	71.75	11.13	72.07	11.23	71.38	11.60	23.58	2.55	23.36	2.58	23.84	2.62
15	10	5	116.1	72.43	10.82	71.38	12.48	74.54	7.14	23.33	2.47	22.98	2.75	24.02	1.87
20	11	9	354.2	70.53	11.67	72.20	11.63	68.49	12.07	23.18	2.68	23.26	2.58	23.08	2.95
21	12	9	618.5	70.20	11.20	70.51	11.04	68.08	12.22	22.98	2.59	22.84	2.35	22.95	3.07
20	12	8	893.8	68.73	10.96	70.04	10.60	66.76	11.92	22.68	2.51	22.70	2.23	22.67	3.05
17	12	5	1,168.2	69.52	10.65	69.83	10.78	68.76	11.56	22.72	2.31	22.62	2.23	22.96	2.77
16	12	4	1,456.9	68.18	10.72	68.93	10.70	65.93	12.09	22.20	2.21	22.33	2.25	21.80	2.35
15	12	3	1,754.0	68.39	10.61	68.65	10.45	67.37	13.59	22.26	2.08	22.24	2.12	22.38	2.34
15	12	3	2,026.1	67.64	10.93	67.94	10.57	66.43	14.78	22.01	2.18	22.01	2.19	22.02	2.65
14	11	3	2,294.2	68.01	10.81	68.19	10.75	67.37	13.47	22.11	2.13	22.03	2.18	22.39	2.35
14	12	2	2,594.5	67.05	10.83	67.44	10.30	64.70	18.53	21.73	2.05	21.85	2.18	20.98	0.81
14	12	2	2,852.0	68.88	9.85	67.66	10.14	76.20	2.12	22.17	2.19	21.92	2.09	23.69	3.06
13	12	1	3,134.2	68.45	9.95	67.73	10.04	77.00		21.90	1.95	21.95	2.03	21.33	
13	12	1	3,401.7	68.58	10.07	67.78	10.06	78.30		21.93	1.91	21.95	1.99	21.69	
12	12		3,724.8	67.35	10.16	67.35	10.16			21.82	2.15	21.82	2.15		
12	12		4,010.8	67.48	9.91	67.48	9.91			21.87	2.06	21.87	2.06		
12	12		4,307.3	66.83	10.50	66.83	10.50			21.64	2.12	21.64	2.12		

BM,body mass; BMI, body mass index; F, finisher; NF, non-finisher; TEFR09, Transeurope Footrace 2009.

**Table 4:** Significance of topographic tissue volume changes regarding percentage volume (vol. %)

	**Mauchly-Test**	**Univariate ANOVA**^**b**^			**Multivariate ANOVA**^**c**^		
**(vol.%)**	***P*-value**	**F value**	***P *value**	**test power**	**F value**	***P *value**	**test power**
**TSV**	0.322	2.565	**0.058**	0.640	-	-	-
**TVV**	0.322	2.565	**0.058**	0.640	-	-	-
**TLT**^a^	0.000	44.605	0.000	1.000	51.592	**0.000**	1.000
**SLT**^a^	0.000	43.573	0.000	1.000	19.556	**0.001**	1.000
**VLT**^a^	0.005	22.980	0.000	1.000	6.699	**0.013**	0.884
**TAT**^a^	0.000	44.655	0.000	1.000	52.762	**0.000**	1.000
**SAST**^a^	0.000	44.721	0.000	1.000	57.109	**0.000**	1.000
**VAT**^a^	0.000	23.718	0.000	1.000	8.598	**0.007**	0.950

^a^mean differences are significant; ^b^‘Greenhouse-Geisser’ correction procedure was used; ^c^Pillai-Spur, Wilks-Lambda, Hotelling-Spur and ‘biggest characteristical root of Roy’. ANOVA, analysis of variance; SAST, somatic adipose soft tissue; SLT, somatic lean tissue; TAT, total adipose tissue; TLT, total lean tissue; TSV, total somatic volume; TVV, total visceral volume; VAT, visceral adipose tissue; VLT, visceral lean tissue.

Bold numbers mark the prefered *P*-value of the prevered ANOVA regarding sphericity assumption proven by Mauchly-Test.

### Percentage body composition

**Figure 5:**
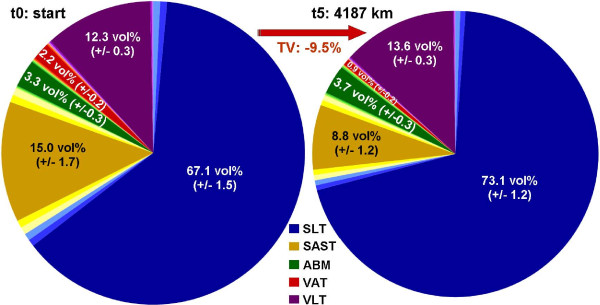
**Adipose and lean volume percentage distribution in finishers at the start and end of TEFR09 (finisher, n**_**F **_**= 12).** TEFR09, Transeurope Footrace 2009, SE is indicated in brackets.

At the start of the TEFR09, the mean percentage volume of TSV was 84.8 (SD 1.36 vol.%). TSV could be differentiated into mean SLT 65.0 vol% (SD 5.33 vol%), mean ABM 3.2 vol% (SD 0.89 vol%) and mean SAST 16.6 vol% (SD 5.58 vol%). The mean TVV of 15.2 vol% (SD 1.36 vol%) is consistent and divides into mean VLT 12.3 vol% (SD 1.23 vol%) and mean VAT 2.9 vol% (SD 1.37 vol%). From these data the changes in mean vol% of tissue compartments regarding the overall population of ultra-runners could be calculated for transcontinental MSUM races (Figure 5).

### Total volumes

**Figure 6:**
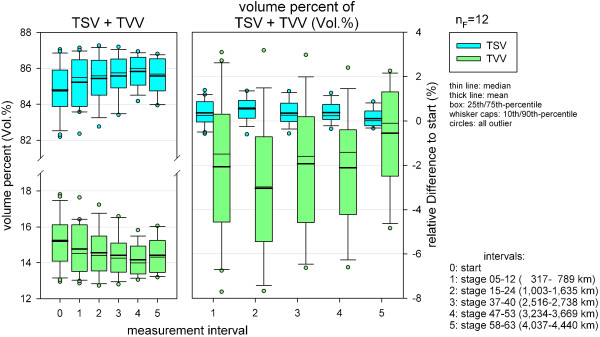
**Changes of somatic and visceral percentage volumes during TEFR09 (finisher, n**_**F **_**= 12).** TEFR09, Transeurope Footrace 2009.

**Figure 7:**
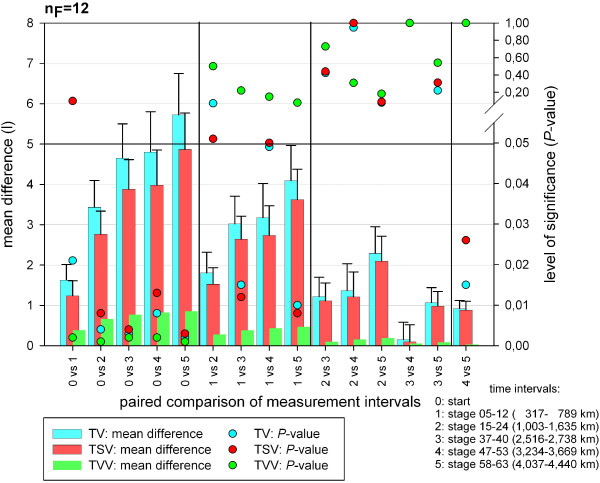
**Post hoc analysis of significance of paired comparison of total volume measurements at different time intervals (finisher, n**_**F **_**= 12).**

Percentage volume changes of TSV and TVV were not significant (Table 3, Figure 6). For absolute volumes (TV, TSV, TVV), however, a significant change could be evaluated with a very high test power (Table 5). Except for TSV at the first measurement interval, significant decreases for TV, TSV and TVV could be shown at all MIs throughout TEFR09 (Figure 7). Paired comparison of MI after the start showed no significant difference for TVV but partial differences for TV and TSV (Figure 7).

**Table 5:** **Significance of topographic tissue volume changes regarding absolute volume measurements (l) (n**_**F **_**= 12)**

	**Mauchly-Test**	**Univariate ANOVA**^**b**^			**Multivariate ANOVA**^**c**^		
**(l)**	***P *value**	**F value**	***P *value**	**test power**	**F value**	***P *value**	**test power**
**TV**^a^	0.000	20.162	0.000	0.999	5.758	**0.020**	0.828
**TSV**^a^	0.001	18.607	0.000	0.999	4.937	**0.030**	0.762
**TVV**^a^	0.000	21.516	0.000	0.999	8.678	**0.007**	0.952
**TLT**	0.516	1.209	**0.322**	0.307	-	-	-
**SLT**	0.516	1.209	**0.322**	0.307	-	-	-
**TAT**^a^	0.000	32.274	0.000	1.000	18.577	**0.001**	1.000
**SAST**^a^	0.000	32.692	0.000	1.000	15.624	**0.001**	0.998
**VAT**^a^	0.000	21.607	0.000	0.999	8.594	**0.007**	0.950

^a^mean differences are significant; ^b^‘Greenhouse-Geisser’ correction procedure was used; ^c^Pillai-Spur, Wilks-Lambda, Hotelling-Spur and ‘biggest characteristical root of Roy.’ ANOVA, analysis of variance; SAST, somatic adipose soft tissue; SLT, somatic lean tissue; TAT, total adipose tissue; TLT, total lean tissue; TSV, total somatic volume; TVV, total visceral volume; VAT, visceral adipose tissue.

Bold numbers mark the prefered *P*-value of the prevered ANOVA regarding sphericity assumption proven by Mauchly-Test.

### Total volume

**Figure 8:**
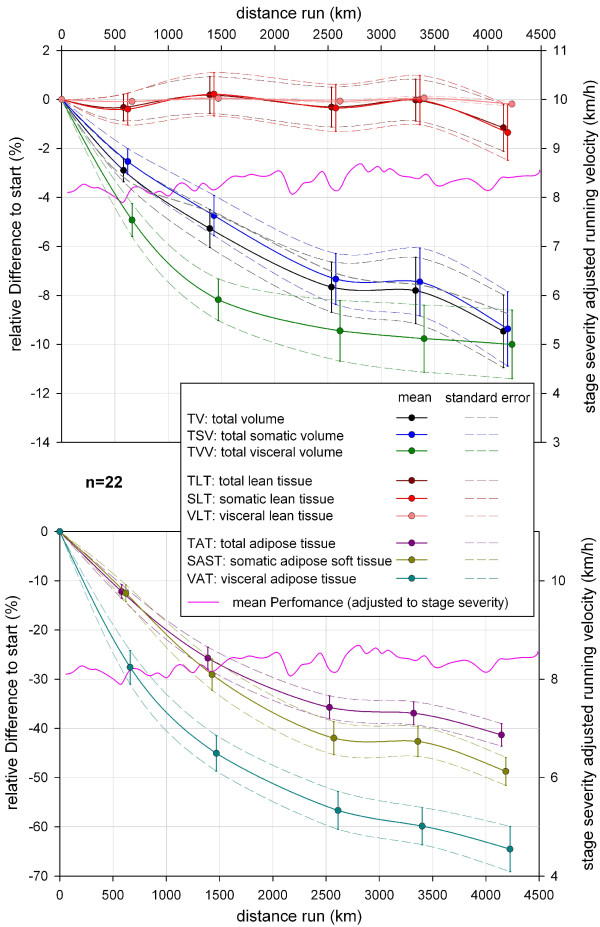
**Mean and standard error of relative changes of specific tissue volume during TEFR09 compared to the start (total sample, n = 22).** TEFR09, Transeurope Footrace 2009.

**Figure 9:**
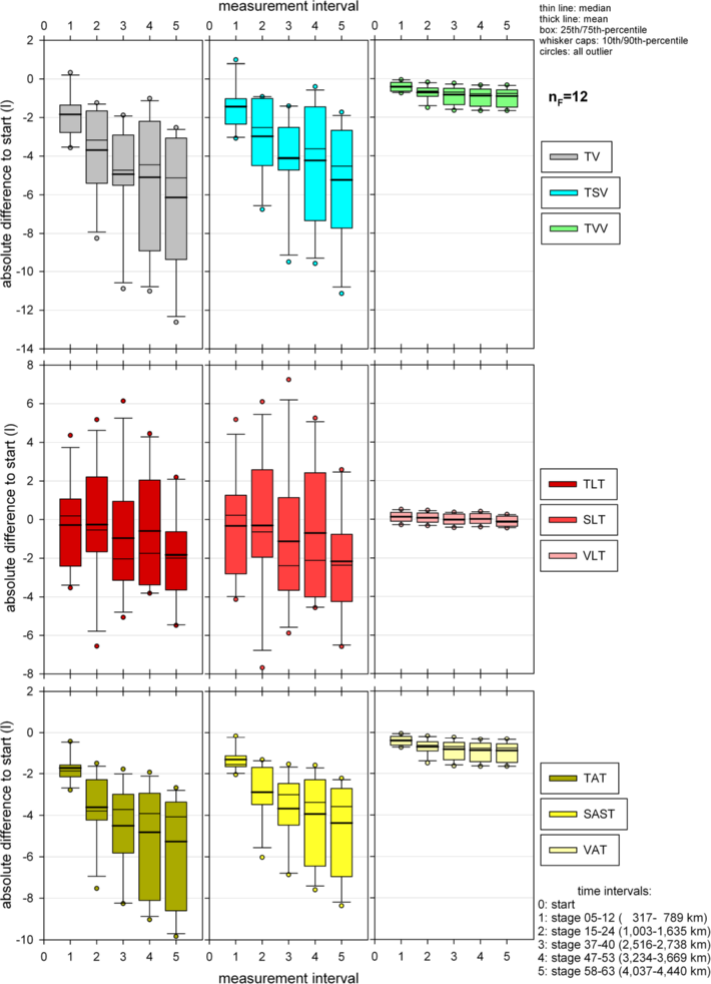
**Absolute changes of specific tissue volume during TEFR09 compared to the start (finisher, n**_**F **_**= 12).** TEFR09, Transeurope Footrace 2009.

**Figure 10:**
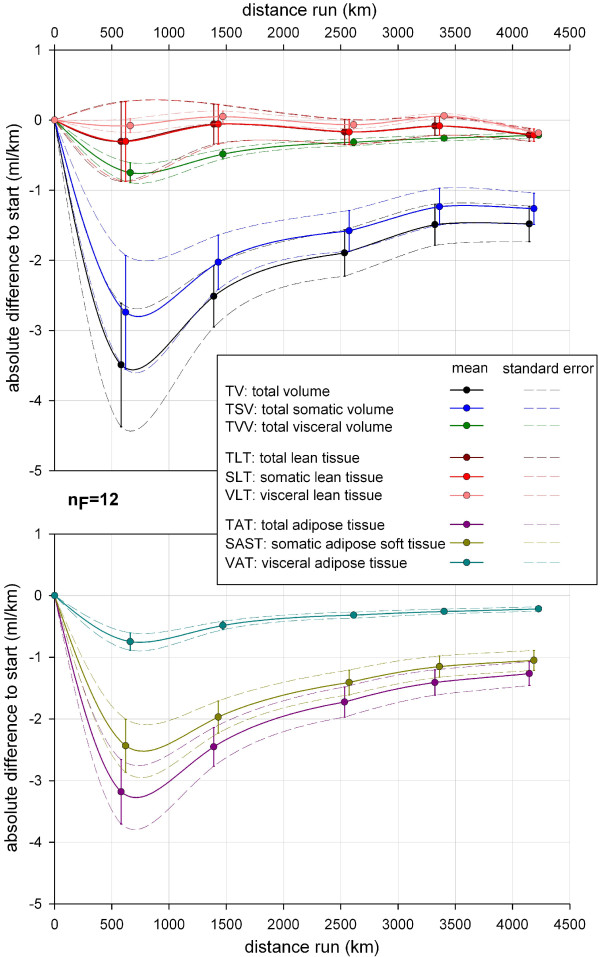
**Absolute volume changes per km compared to the start in the finisher group (finisher, n**_**F **_**= 12).** TEFR09, Transeurope Footrace 2009.

After more than 4,000 km of running the mean TV showed a mean decrease of 9.5% (SD 5.1%, min −2.7%, max −17.9%) compared to the start. Depending on the total sample the mean TV decrease for the overall population of ultra-runners ranges between 8% to 11% (SE 1.5%), (Figure 8). Looking only at the group of finishers, the absolute amount of mean TV loss at the last MI was 6.1 L (SD 3.4 L, min−2.5 L, max −12.6 L (Figure 9). Mean loss of TV per km was 3.5 ml (SD 2.9 ml/km) in the beginning and became smaller, like a reversed parabolic function, during TEFR09 down to 1.5 ml/km (SD 0.8 ml/km) at the end of the race (Figure 10).

### Total somatic volume

The relative decrease of TSV during TEFR09 showed a nearly similar curve as TV (Figure 8), but was less pronounced (mean −9.4% after more than 4,000 km, SD 5.3%, min −2.1%, max −18.1%) with the same SE of 1.5%. For the finisher group the absolute loss of TSV increased to 5.2 L in the mean (SD 3.0 L, min −1.7 L, max −11.1 L) at the end of the race (Figure 9). This is consistent with a mean TSV loss of 1.3 ml/km (SD −0.7 ml/km) at the end of TEFR09, starting with 2.7 ml/km (SD 2.7 ml/km) in the first eight stages of TEFR09 (Figure 10).

### Total visceral volume

Compared to TV and TSV, the relative decrease of TVV occurred much faster but ended in a nearly similar amount with a mean of 10.0% (SD 4.9%, min −3.8%, max −19.3%) in a negative parabolic graph shape (see Figure 8). The mean loss of absolute TVV was 0.9 L (SD 0.5 L, min −0.3 L, max −1.7 L) for finishers (Figure 9). Mean absolute TVV loss per km during TEFR09 had a maximum of 0.75 ml/km (SD 0.5 ml/km) at the beginning and 0.2 ml/km (SD 0.1 ml/km) at the end (Figure 10).

### Adipose tissue

**Figure 11:**
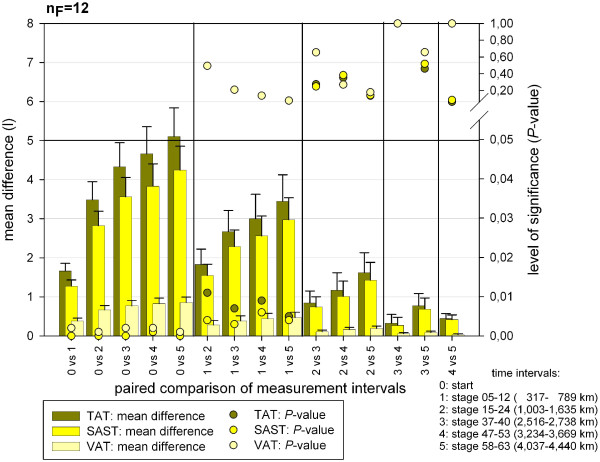
**Post hoc analysis of the significance of the paired comparison of total fat tissue measurements at different time intervals (finisher, n**_**F **_**= 12).**

In total (TAT), somatic (SAST) and visceral (VAT) adipose tissue, a significant change of absolute volumes (Table 5) and percentage volumes (Table 3) could be evaluated with a very high test power at the different MIs during TEFR09. A significant decrease for TAT, SAST and VAT could be shown at all MIs throughout TEFR09 compared to the start and for TAT and SAST compared to the first MI after the start (stage 5 to 12) (Figure 11). For other MIs the paired comparison showed no significant change.

### Total adipose tissue

There was a continuous decrease of TAT that ended in a relative mean loss of 41.3% (SD 8.0%, min −25.4%, max −53.2%) with a small SE of 2.3% (Figure 8) at the last MI. Looking at the absolute loss of TAT, a finisher lost 5.3 L in the mean (SD 2.6 L min −2.7 L, max −9.8 L) until the end of the race (Figure 9). The mean TAT loss per km in finishers was 3.2 ml (SD −1.7 ml/km) at the beginning and 1.2 ml (SD 0.6 ml/km) at the end of TEFR09 (Figure 10).

### Somatic adipose soft tissue

The relative SAST decrease compared to the start showed a steeper graph than TAT and ended in a mean loss of 48.7% (SD 9.9%, min −25.9%, max −65.5%) after more than 4,000 km (Figure 8). The absolute SAST decrease in finishers showed a mean of 4.4 L (SD 2.2 L, min −2.2 L, max −8.4 L) at the end of TEFR09 (Figure 9). This corresponds to a mean loss of SAST of 1.1 ml/km (SD 0.5 ml/km) at the end of TEFR09 compared to 2.4 ml/km (SD 1.4 ml/km) at the start (Figure 10).

### Visceral adipose tissue

**Figure 12:**
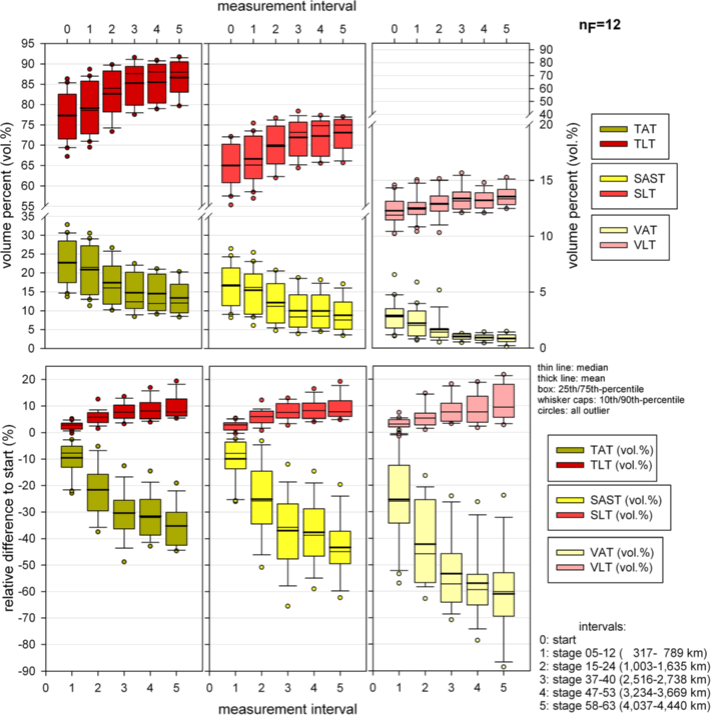
**Changes of adipose and lean tissue percentage volumes during TEFR09 (finisher, n**_**F **_**= 12).** TEFR09, Transeurope Footrace 2009.

The relative decrease of VAT occurred much more rapidly in the mean and ended in a relative VAT volume loss of 64.5% (SD 15.9%, min −27.7%, max −88.8%) at the end of the race (Figure 8) compared to the start and a SE up to 4.6%. The percentage volume of VAT decreased more quickly and severely compared to absolute VAT volume (Figure 12). In absolute values, this rapid and continuous loss of VAT ended in a mean of −0.9 L (SD 0.5 L, min −0.3 L, max −1.7 L) in the finisher group (Figure 9), which was nearly the same as the absolute TVV loss. Therefore, the mean VAT volume loss per km was the same as for TVV in the finishers (Figure 10).

**Figure 13:**
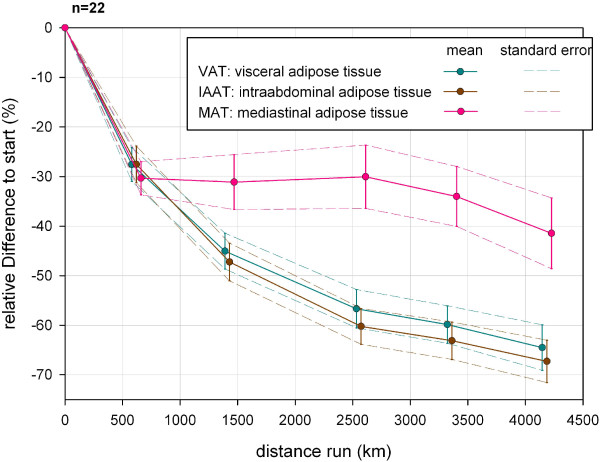
**Relative changes of visceral adipose volume during TEFR09 compared to the start (total sample, n = 22).** TEFR09, Transeurope Footrace 2009.

The subdivision of VAT into IAAT and MAT shows that IAAT decreased a bit faster than VAT in total and ended in a relative volume loss of 67.3% (SD 14.8%, min −31.7%, max −88.8%) at the end (Figure 13). MAT initially decreased as rapidly as IAAT with respect to VAT but reached a plateau of 30% volume loss after nearly 1,000 km of running before it decreased again in the last third of the race up to 41.5% with a larger variance (SD 24.7%, min −0.1%, max −89.0%).

### Lean tissue

Due to significant and continuous loss of different adipose tissue volumes the percentage volume of TLT, SLT and VLT increased during TEFR09 significantly without relevant changes in absolute volumes, respectively (Table 3, Figure 12); analysis of the means of absolute volume showed no significant changes for total, somatic and visceral lean tissue at the different MIs during TEFR09 (Table 5).

### Total lean tissue, somatic lean tissue, visceral lean tissue

TLT volume showed undulating relative changes duringTEFR09 in the mean compared to the start. Just at the end, after more than 4,000 km running, the mean relative changes were −1.2% TLT (SD 3.3%, min 6.3%, max −5.5%) with an SE of 1.0% (Figure 8). Due to nearly stable volume regarding VLT during TEFR09, the SLT data during TEFR09 was similar to that of TLT relative to the start: mean −1.4% (SD 3.9%, min 7.5%, max −6.6%). Not every finisher showed a decrease of absolute TLT and SLT; some of them showed increases, some decreases: mean −0.9 l (SD 1.2 L, min 1.1 L, max −2.8 L); see Figure 9. Mean loss of TLT and SLT per km changed between 0.3 and 0.2 ml with a wide range (SD at beginning 1.9 ml/km, at the end 0.3 ml/km), see Figure 10.

### Segmental volume analysis

**Figure 14:**
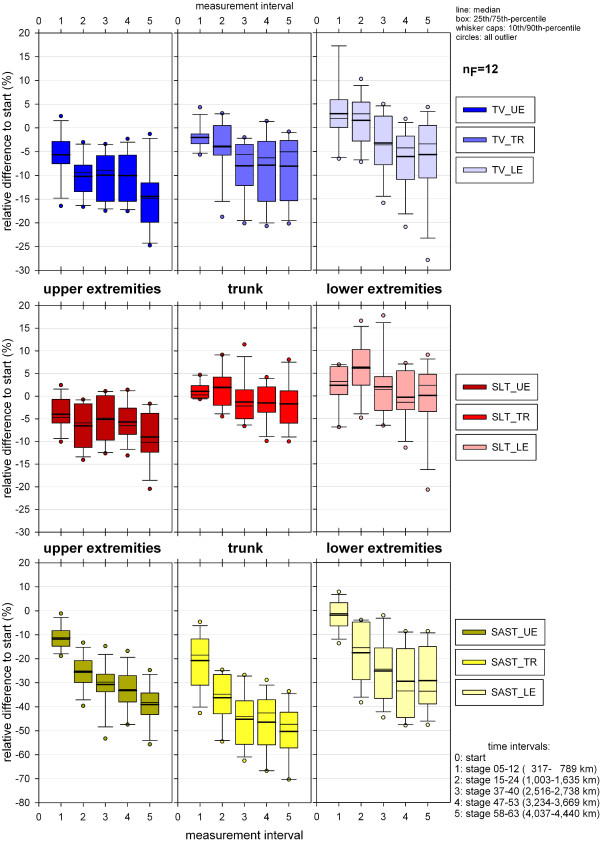
**Relative changes of segmented tissue volume (UE, TR, LE) during TEFR09 compared to the start (finisher, n**_**F **_**= 12).** LE, lower extremities; TEFR09, Transeurope Footrace 2009, TR, trunk; UE, upper extremities.

The significance of the volume changes in the different body segments is shown in Table 6. For the lower extremities the change of volumes was only significant for SAST_LE but not for TV_LE or LT_LE; for the trunk and upper extremities decreases were significant for adipose soft tissue volume (SAST_TR, SAST_UE) and total volume (TV_TR, TV_UE) but not for lean tissue volume (LT_TR, LT_UE). Most decrease of somatic adipose tissue occurred in the trunk (t5: mean −50.3%, SD 12.0%), followed by the arms (t5:mean −39.1%, SD 8.3%); in the legs the adipose tissue lose was the smallest, but significant (t5: mean −29.2%, SD 13.4%), Figure 14. Although changes of lean tissue were not significant in any segment, mean values demonstrate a mean increase in the legs in the first half of TEFR09, and in the trunk in the first third of the race, while in the arms lean tissue loss was already detectable at the first MI t1 (Figure 14).

**Table 6:** **Significance of segmental volume changes regarding repeated absolute volume (l) measurements (n**_**F **_**= 12)**

	**Mauchly-Test**	**Univariate ANOVA**^**b**^			**Multivariate ANOVA**^**c**^		
**(l)**	***P *value**	**F-value**	***P *value**	**test power**	**F-value**	***P *value**	**test power**
**TV_LE**	0.003	7.763	0.002	0.946	2.341	**0.149**	0.423
**TV_TR**^a^	0.133	6.349	**0.003**	0.918	-	-	-
**TV_UE**^a^	0.001	27.504	0.000	1.000	13.942	**0.002**	0.996
**SLT_LE**	0.003	6.411	0.20	0.733	14.587	**0.095**	0.597
**SLT_TR**	0.252	3.534	**0.21**	0.769	-	-	-
**SLT_UE**	0.700	3.128	**0.29**	0.734	-	-	-
**SAST_LE**^a^	0.000	63.294	0.000	1.000	20.644	**0.000**	1.000
**SAST_TR**^a^	0.000	17.388	0.000	0.996	13.387	**0.002**	0.995
**SAST_UE**^a^	0.000	16.151	0.000	0.987	3.389	**0.041**	0.584
**TV_LE**^a^	0.001	27.504	0.000	1.000	13.942	**0.002**	0.996
**TV_TR**^a^	0.133	6.349	**0.003**	0.918	-	-	-
**TV_UE**	0.003	7.763	0.002	0.946	2.341	**0.149**	0.423

^a^mean differences are significant; ^b^‘Greenhouse-Geisser’ correction procedure was used; ^c^Pillai-Spur, Wilks-Lambda, Hotelling-Spur and ‘biggest characteristical root of Roy’. ANOVA, analysis of variance; LE, lower extremities; SAST, somatic adipose soft tissue; SLT, somatic lean tissue; TR, trunk; TV, total volume; UE, upper extremities.

Bold numbers mark the prefered *P*-value of the prevered ANOVA regarding sphericity assumption proven by Mauchly-Test.

### Finisher/non-finisher

**Figure 15:**
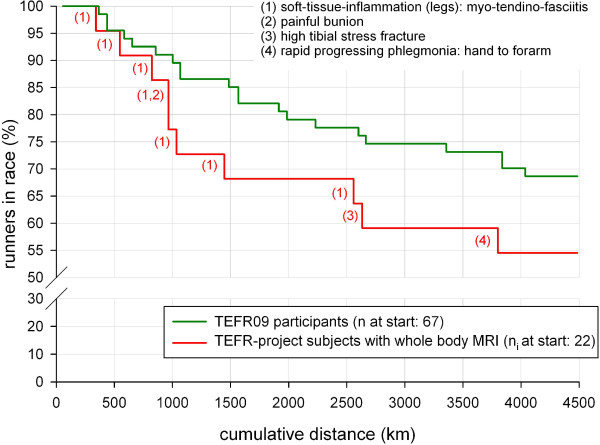
Dropout rate.

A total of 45.5% of the subjects did not finish the race. The dropout rate of subjects compared to all race participants is shown in Figure 15. The main reason (70%, n_i_ = 7) for premature dropping out of the race was intolerable pain in the legs due to an overload of muscle and tendons (soft tissues) leading to intermuscular and peritendinous inflammation (fasciitis): lower legs (40%), upper legs (30%). Other reasons were a high tibial stress fracture, a painful bunion and one rapidly progressing phlegmonia from the thumb up to the forearm needing immediate surgical intervention.

**Figure 16:**
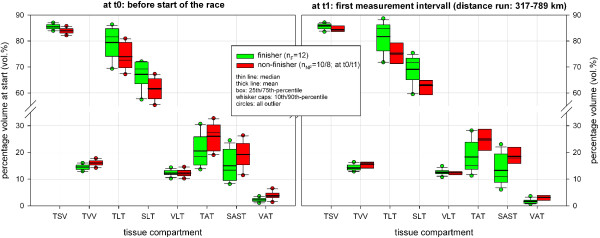
**Difference between F and NF regarding percentage tissue volumes before the start of TEFR09 and at MI t1.** F, finisher; measurement interval; NF, non-finisher; TEFR09, Transeurope Footrace 2009.

**Figure 17:**
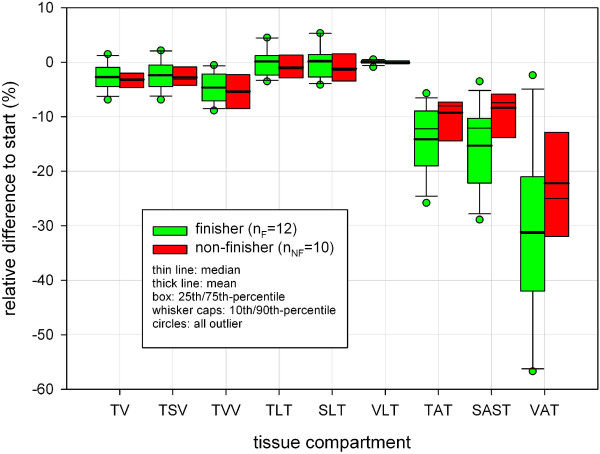
**Difference between F and NF regarding relative volume changes of tissue compartments at first measurement interval (t1) of TEFR09.** F, finishers; NF, non-finishers; TEFR09, Transeurope Footrace 2009.

Figure 16 shows the distribution of percentage volumes for all tissue compartments at the start time (t0) and MI t1 (317 to 789 km) for finishers (n_F_ = 12) and non-finishers (n_NF_ = 10) of TEFR09. At both times the finisher group had significantly more percentage volume regarding total somatic tissue (mean TSV) than non-finishers of TEFR09 (at t0 +1.8%: 85.5 vol% versus 84.0 vol%, at t1 +1.6%: 85.8% versus 84.4%) and, therefore, significantly less percentage volume of mean TVV (at t0 -10.5%: 14.5 vol% versus 16.0 vol%, at t1 -9.5%: 14.2 vol% versus 15.6 vol%), Table 7. The finisher group showed significantly less adipose tissue volume percentage than the non-finishers for TAT and VAT at t0 and t1, and also for SAST at t1 (Table 7). At the start, non-finishers had 71.5% more VAT volume percent (mean VAT at t0: 2.2 vol% versus 3.8 vol%), 28.0% more SAST volume percent (mean SAST at t0: 15.0 vol% versus 19.2 vol%) and in total 26.6% more TAT volume percent (mean TAT at t0: 20.6 vol% versus 26.1 vol%) than the finishers (Table 7). At the first MI, t1, the difference between finisher and non-finisher was significantly further on; non-finishers had 96.8% more VAT volume percent (mean VAT at t0: 1.6 vol% versus 3.2 vol%), 39.7% more SAST volume percent (mean SAST at t0: 13.3 vol% versus 18.5 vol%) and in total, 34.9% more TAT volume percent (mean TAT at t0: 18.3 vol% versus 24.7 vol%) than finishers (Table 7). These differences for adipose tissue compartments were no longer detectable as the race proceeded (t2 to t5); either there are not enough numbers to treat in the group of non-finishers for further analysis on the difference with finishers or no difference could be shown. Conversely, the lean tissue difference of percentage volume was significantly smaller in non-finishers compared to finishers for TLT (at t0: -6.9%, at t1: -7.8%) and SLT (at t0: -8,1%, at t1: -8.9%) (Table 7). VLT showed no significant difference between finishers and non-finishers at any MI (t0 to t5). Table 8 and Figure 17 demonstrate a significant relative volume loss at MI t1 and t2 compared to the start only for SAST and no other tissue compartment.

**Table 7:** Analysis of the difference of percentage volume (vol%) between F/NF at the start (t0) and MI (t1, t2) for total, lean and adipose tissue compartments

	**t0: n**_**F **_**= 12, n**_**NF **_**= 10**		**t1: n**_**F **_**= 12, n**_**NF **_**= 8**		**t2: n**_**F **_**= 12, n**_**NF **_**= 6**	
	**Mean difference of percentage volume (vol.%)**	***P *value (ITT)**	**Mean difference of percentage volume (vol.%)**	***P *value (ITT)**	**Mean difference of percentage volume (vol.%)**	***P *value (ITT)**
**TSV**	1.52	**0.005**^**a**^	1.35	**0.036**^**a**^	1.07	0.182
**TVV**	−1.52	**0.005**^**a**^	1.35	**0.036**^**a**^	1.07	0.182
**TLT**	4.04	**0.031**^**a**^	6.39	**0.015**^**a**^	5.05	0.088
**SLT**	4.16	**0.014**^**a**^	6.18	**0.010**^**a**^	4.65	0.058
**VLT**	0.032	0.953	0.21	0.707	0.39	0.604
**TAT**	−5.47	**0.031**^**a**^	−6.39	**0.015**^**a**^	−5.04	0.088
**SAST**	−4.21	0.080	−5.27	**0.032**^**a**^	−3.64	0.184
**VAT**	−1.60	**0.006**^**a**^	−1.56	**0.021**^**a**^	−1.46	0.060

^a^mean differences between finishers and non-finishers are significant. t0: at start of TEFR09, t1: stage 5 to 12 (317–789 km), t2: stage 15 to 24 (1,003 to 1,635 km). F, finishers; ITT, t-test for independent samples; MI, magnetic imaging; NF, non-finishers; SAST, somatic adipose soft tissue; SLT, somatic lean tissue; TAT, total adipose tissue; TERF09, Transeurope Footrace 2009; TLT, total lean tissue; TSV, total somatic volume; TVV, total visceral volume; VAT, visceral adipose tissue; VLT, visceral lean tissue.

Bold numbers mark significant *P*-values.

**Table 8:** Analysis of the difference in relative volume changes (%) at MI t1 and t2 compared to start between F/NF for total, lean and adipose tissue compartments

	**t1 versus t0: n**_**F **_**= 11, n**_**NF **_**= 9**		**t2 versus t0: n**_**F **_**= 11, n**_**NF **_**= 7**	
	**Mean diff. of relative changes (%)**	***P *value (ITT)**	**Mean diff. of relative changes (%)**	***P *value (ITT)**
**TV**	0.47	0.608	−1.49	0.306
**TSV**	0.41	0.690	−2.06	0.190
**TVV**	0.69	0.640	1.35	0.410
**TLT**	1.09	0332	−0.18	0.913
**SLT**	1.34	0.319	−0.21	0.917
**VLT**	0.07	0.923	−0.24	0.545
**TAT**	−4.86	0.086	−9.52	0.078
**SAST**	−6.93	**0.031**^**a**^	−14.27	**0.046**^**a**^
**VAT**	−8.92	0.191	−8.88	0.276

^a^mean differences between finishers and non-finishers are significant. F, finishers; ITT, t-test for independent samples; measurement interval; NF, non-finishers; SAST, somatic adipose soft tissue; SLT, somatic lean tissue; t0, at start of TEFR09; t1,stage 5 to 12 (317 to 789 km); t2, stage 15 to 24 (1,003-1,635 km); TAT, total adipose tissue; TEFR09, Transeurope Footrace 2009; TLT, total lean tissue; TSV, total somatic volume; TV, total volume; TVV, total visceral volume; VAT, visceral adipose tissue; VLT, visceral lean tissue.

Bold numbers mark significant *P*-values.

### Prerace performance

**Figure 18:**
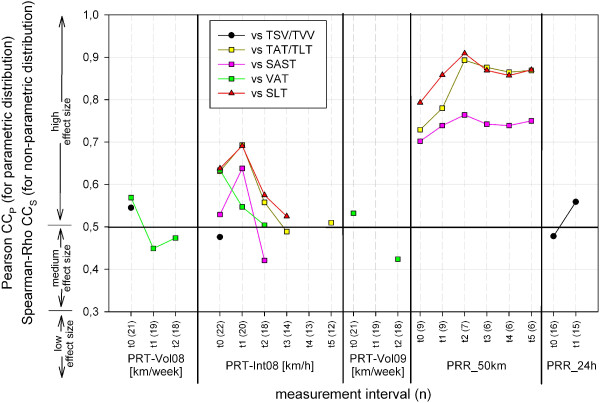
Correlation (one-tailed test) of percentage volumes and prerace performance.

Although there is a wide range of finished long distance foot races in the subject group (Table 9), every participant of TEFR09 had already finished nearly one UM and MSUM, but not every subject had finished a single marathon. The endurance training extent of one year and three months before TEFR09 also varied for training volume (km), time (hours) and intensity (km/hour) in the subject group (Table 9). For the number of finished marathons, UM and MSUM no difference between finisher and non-finisher could be evaluated (Table 10). However, regarding prerace training volume and intensity one year before TEFR09 and their 50 km- and 24 hour-race record, finishers had a significantly higher prerace-performance compared to non-finishers (Table 10). Only these five prerace-performance parameters (PRT_Vol08,_ PRT_Vol09_, PRT_Int08,_ PRR_50km_, PRR_24hr_) also showed a mainly high and medium correlation with the volume percentage of adipose tissue compartments (VAT, SAST, TAT), TLT and SLT (Figure 18).

**Table 9:** Endurance running history of subjects (n = 22)

	**Mean**	**SD**	**95%-percentile**	**Range**
Years of regular endurance running	17.4	7.6	6.3 to 31.8	6 to 32
M finished (number)	123,1	218,2	2 to 297.8	2 to 988
UM finished (number)	90,8	68	11.3 to 248.9	11 to 255
MSUM finished (number])	6,3	2,9	1.3 to 13.6	1 to 14
**Endurance training extent 2008 (one year before TEFR)**				
Annual running distance (km/year)	5,468	1,720	3,000 to 9,000	2,580 to 9,152
PRT08 volume (km/week)	105.1	32.4	50.8 to 175.4	50 to 176
PRT08 time (hours/week)	12.5	3.1	7.1 to 19.6	7 to 20
PRT08 intensity (km/hour)	8.3	1.5	6.5 to 10.9	7 to 11
**Endurance training extent last two months before TEFR**				
Total running distance ([km)	898	267	500 to 1260	500 to 1,500
PRT09 volume (km/week)	110.5	33.8	60.5 to 186	60 to 190
PRT09 time (hours/week)	13.2	3.5	8 to 21.6	8 to 22

M, marathon; MSUM, multistage ultramarathon; PR, prerace; TEFR, Transeurope Footrace; UM, ultramarathon.

**Table 10:** Distribution type and analyses of the difference between F/NF regarding prerace performance indices

	**Values**			**Mean (SD)**		**Test on normal distribution Shapiro-Wilk **[29]		**Test on difference F/NF**	
	**Valid**	**Missing**							
	**n**	**n**	**(%)**	**F**	**NF**	**statistic**	***P***	**test type**^**a**^	***P ***^**b**^
PRY (years)	21	1	4.5	16.5	18.7	0.936	0.185^c^	ITT	0.530
n_F_ M (n)	19	3	13.6	81.5	194.3	0.481	0.000	MWU	0.211
n_F_ UM (n)	22	0	0	94.8	86.0	0.858	0.005	MWU	0.895
n_F_ MSUM (n)	22	0	0	7.0	5.4	0.936	0.165^c^	ITT	0.146
PRT_Vol08_ (km/week)	21	1	4.5	**117.9** (34.3)	**88.0** (20.8)	0.959	0.505^c^	ITT	**0.032**^**d**^
PRT_Time08_ (hour/week)	21	1	4.5	13.0	11.9	0.971	0.761^c^	ITT	0.427
PRT_Int08_ (km/hour)	22	0	0	**9.0** (1.47)	**7.5** (0.93)	0.909	0.044	MWU	**0.040**^**d**^
PRT_Vol09_ (km/week)	21	1	4.5	**126.1** (35.2)	**89.7** (17.4)	0.956	0.435^c^	ITT	**0.010**^**d**^
PRT_Time09_ (hours/week)	21	1	4.5	14.2	12.0	0.947	0.303^c^	ITT	0.159
PRR_M_ (hours)	15	7	31.8	3.1	3.1	0.851	0.018	MWU	0.676
PRR_50km_ (hours)	9	13	59.1	**4.3** (0.57)	**5.1** (0.26)	0.914	0.343^c^	ITT	**0.026**^**d**^
PRR_100km_ (hours)	17	5	22.7	9.5	10.4	0.933	0.248^c^	ITT	0.241
PRR_6hr_ (km)	13	9	40.9	67.9	59.7	0.918	0.238^c^	ITT	0.218
PRR_12hr_ (km)	10	12	54.5	99.9	87.3	0.940	0.548^c^	ITT	0.558
PRR_24hr_ (km)	16	6	27.3	**199.8** (22.5)	**168.5** (26.0)	0.961	0.672^c^	ITT	**0.036**^**d**^

^a^ITT: independent t-test; ^b^bilateral (asymptotic) test; ^c^significance not shown (*P* >0.05): normal distribution accepted; ^d^mean differences between finishers and non-finishers are significant. F, finishers; M, marathon; MSUM, multistage ultramarathon; MWU: Mann–Whitney-U test; n, number; NF, non-finishers; PRR, prerace records; PRT, prerace training; PRY, years of regular endurance running; IM, ultramarathon.

Bold numbers mark significant *P*-values of the test on difference between F and NF.

### Race-performance

**Figure 19:**
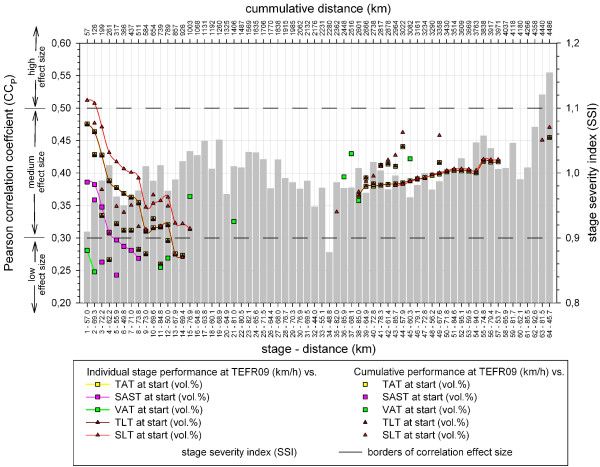
**Correlation of adipose and lean volumes at start with performance at TEFR09.** TEFR09, Transeurope Footrace 2009.

No relevant correlation between percentage fat and lean volumes of different compartments at the start and the race performances of the subjects at TEFR09 could be detected (Figure 19). For SAST at the beginning of TEFR09 (stage 1 to 8) a significant correlation between percentage volume at the start and cumulative performance is given, but only at a medium to low effect size. For TAT, TLT and SLT the significance for such a correlation is shown at the first 12 to 15 stages and during the last third of TEFR09 at a medium effect size (Figure 19). A correlation of percentage fat and lean volumes to performance at the individual stages can only be shown for a few stages at a middle to low effect size. None of the relative changes in the investigated volumes duringTEFR09 were significantly correlated with performance.

## Discussion

Almost nothing is known about the influence of the endurance burden on the specific changes in body composition regarding distribution of adipose and lean tissues in somatic and visceral compartments and in the body segments. Field studies on this topic mostly use methods which only allow indirect measurements and approximated calculations or simple estimations of total or local adipose or lean tissue proportions [11-14]. For TAT and subcutaneous adipose tissue (SCAT = SAST without intermuscular adipose tissue (IMAT) [31]), some of these indirect methods show more or less correlation to MRI findings [17]. These methods are not able to predict the amount of visceral (VAT) or somatic adipose tissue (SAT) in the body [16,32]. Being the first investigation in endurance field studies using the gold standard method [18] whole body MRI for such analyses, our results provide new data on the volume changes of fat and lean tissue in these different parts of the athlete’s body.

### Age and gender related differences

Bale *et al*. [33] found a lower percentage of body fat in female elite marathon runners. In obese patients (BMI >27 kg/m^2^) Machann *et al*. [25] found that the amount and distribution of adipose tissue correlated with age (VAT increasing with age) and with gender (%SAT female > male, %VAT male > female). They found no consistent differences in TAT profiles between the selected age groups for both females (n = 40, mean age 45 years, SD 12 yrs., range 23 to 64 yrs.) and males (n = 40, mean age 45years SD 12 yrs., range 24 to 65 yrs.) in their group. Naturally, our group of ultra-runners with a comparable age distribution (n = 22, mean age 49 years, SD 12 years, range 27 to 69 years) showed a very low absolute mean volume of VAT at the start of TEFR09 (females: 0.5 L, males 1.8 L) compared to obese patients (females 1.5 to 4 L, males 4 to 6.8 L) [25]. Statistical analysis of gender-related differences was not possible (only two females) in our group, but even these data indicate that a difference in VAT between men and women is not only visible in obese people, but is also visible in thin ultra-endurance athletes. Analysis based on age showed no correlation to fat distribution at the start (TAT, SAST, VAT) or to volume changes of lean and adipose tissue during TEFR09.

### Changes in body composition

Different effects of endurance performance on body composition are described in the literature. Beyond dispute is the fact that endurance performance leads to a decrease in body mass, mainly body fat. Body fat is the main energy-rich substrate for endurance performance [34-37]. Therefore, endurance exercise leads to a reduction of subcutaneous tissue as demonstrated in several field studies [34,36,38].

The specific influence on energy turnover seems to depend on the type of endurance burden [1,39]. In general, non-stop ultra-endurance races over hours, days or weeks without a break result in a decrease in body mass [1,36,40,41] in which body fat as well as skeletal muscle seems to decrease [1,36,40-42]. In ultra-endurance performances with defined breaks, body mass may remain stable [43-45] or even increase [34] and body fat is reduced [34,46,47], whereas skeletal muscle mass seems to be spared [35,43,47] or may even increase [46]. Our whole body MRI results show comparable results for an ultra-long MSUM over 64 days without any day rest; every subject decreased in BM(I), TV, TSV and TVV due to massive loss of TAT, SAT and VAT, respectively. Not every runner lost TLT and SLT during the TEFR09. Some of them showed increases, some decreases. Knechtle *at al*. found the same individual differences for lean tissue in ultra runners during a 1,200 km MSUM across Germany [48]. If there are not sufficiently long breaks in ultra-endurance races, some participants might not find enough time for regeneration and restoration of their energy depots before the next stage. As the race progresses this leads to the utilization of muscle tissue for energy provision.

### Mass loss

Raschka and Plat observed a mean loss of 1.75 kg body mass in an ultra-endurance run over 1,000 km within 20 days [34]. In their investigation, there was a statistically significant decrease in body mass after day 8 until day 11, which then remained stable until the finish. In another investigation of 10 ultra runners (BIA), the mean loss of BM after a 1,200 km footrace was also not significant, but the loss of 3.9 kg fat mass was [48]. Unfortunately, the authors gave no information about the relative changes of fat and lean body mass. Our results determined that a transcontinental ultra-long MSUM of 64 stages leads to a significant three times higher loss of body volume (9.5%) than published for body mass loss in deca-triathlons or 20 stage MSUMs [34,47].

The relation of water and lipid to the density of human adipose tissue ranges from 0.925 to 0.97 kg/L [49]. Assuming the middle value (0.948 g/L), in our investigation the ultra-athletes lost a total fat mass (TAT) of 4.8 kg in the mean (SAST 4.0 kg, VAT 0.8 kg), resembling the main part (91.8%) of body mass loss of 5.2 kg. The lean tissue of the human body has a higher density than adipose tissue and muscle tissue (range 1.05 to 1.06 g/L) and varies with age [49,50], ranging between 1.10 and 1.11 g/L [51,52]. With these data and knowing the mean relative reduction of TLT (1.2%), the mean loss of lean body mass can be calculated as about −0.67 kg at the end of TEFR09 in our subject group.

### Visceral adipose tissue

Mediastino-abdominal lipomatosis is described as being associated with exertional dyspnea [53], non–insulin-dependent diabetes, type IV hyperlipidemia, and hyperuricemia. The abdominal VAT is an important independent risk factor for metabolic diseases in the older patient [54] and there is evidence that mainly abdominal VAT, which is morphologically and functionally different from abdominal SAST, is associated with the metabolic syndrome (insulin resistance, dyslipidemia, hypertension, obesity) and hyperinsulinemia [55-60], as well as linked inflammatory diseases [61]. The real mean loss of relative IAAT while running a MSUM of nearly 4,500 km, was more than two thirds compared to the start in our group (Figure 13). We showed that endurance running also has an direct influence on intrathoracic fat, especially MAT, which decreased up to more than 40% in the mean (Figure 13). MAT is associated with hypertension, obesity, and iatrogenic Cushing syndrome [57,62-65].

Until now, a specific treatment for the selective reduction of VAT is not known [66] and as our MR analyses showed that VAT decreased much more rapidly and vigorously than SAST (Figure 8), a very good and effective way to reduce the risk of metabolic disease is endurance running. As VAT decreases much faster and more than SAST, our investigation indicates that three-compartment measurement methods, such as SF-analyses and BIA, cannot give accurate assumptions or calculations for IAAT and MAT. Even the results of the four-compartment method cadaver study are false, when post mortem findings are transferred to physiological effects which occur from the impact of long lasting running on fat and lean tissue in vivo [67].

### Finishers versus non-finishers

55% (n = 12) of the 22 ultra-runners treated with mobile whole body MRI for this study reached the last measurement interval; 10 dropped out earlier. In contrast, the dropout rate for all starters at TEFR09 and all subjects taking part in the TEFR project was 31% [2]. Reasons for dropping out of this transcontinental MSUM race were overuse reactions of the musculoskeletal system of the lower extremities (80%, Figure 15), mainly concerning the myotendinous fascial system.

In a 17-day MSUM (1,200 km) Knechtle et al. found no differences between finishers and non-finishers regarding the anthropometric parameters, BMI, SF, CF, estimated skeletal muscle mass (estimated from SF and CF) and percent body fat (BIA) [68]. With whole body MRI for differentiated body composition analysis, however, we found significant differences between finishers and non-finishers between both somatic and visceral volumes and between adipose and lean tissue volumes at the start and early beginning of the 4,500 km MSUM TEFR09 (Figure 16). Out results indicate that the risk of dropping out of such an ultra-long transcontinental footrace is significantly higher when the total body fat percentage is more than 21% to 25% at the start, in which the visceral fat percentage (VAT) shows a higher difference between finishers and non-finishers (71.5% in the mean) than the somatic fat compartment (SAST, 28.0%). Because VAT is affected by the endurance running burden most quickly and most deeply compared to somatic fat and other lean tissue (Figure 9) and is highly correlated with prerace performance regarding training volume and intensity and specific ultramarathon race-performance (50 km-race), our results indicate,that VAT is the most sensible predictor for the risk of non-finishing a transcontinental MSUM, such as the TEFR09. In ultra-runners there is not a high SAST or TAT, if the VAT is low.

Although training a distance of 4,500 to 5,000 km is not possible, participants of such MSUMs should acquire specific characteristics and levels regarding body composition and performance skills even before the race if they want to have a good chance to finish: VAT near 20% to 21%, training volumes of more than 100 km/week one year before the race and the performance intensity of 7.5 km/hour at a minimum allowing specific ultra-race records of less than 5 hours in 50 km-races or more than 178 km in 24-hour races. In other words, if these levels of prerace performance are reached for at least 15 months before the transcontinental race, the VAT (and SAST, TAT) as the sensible marker for specific body composition adaptation is also in an optimal range for low risk of non-finishing, because these parameters correlate in a mostly high level.

Because the subjects mainly fall out of the race due to overuse injuries in the myotendinous fascial system of the lower extremities, we tend to assume that the mentioned interdependent parameters of body composition and prerace ultra-running performance, lead to overuse injuries in the main stressed musculoskeletal organs, if they are not highly adapted as mentioned above; too little specific ultra endurance adaption and too much of VAT (and SAST) results in a high risk of severe soft tissue overuse in the legs and mostly happens in the early phase (Figure 15) of a transcontinental foot race.

Nearly every starter of TEFR09 showed, more or less often, overuse soft tissue problems of the myotendinous fascial structures of the legs during the race, but the feet are not a region for problems for experienced endurance runners in a MSUM [69]. So the immense amount of mechanical stress on the musculoskeletal system when running nearly two marathons daily over a period of nine weeks can lead to these overuse syndromes without the obligatory necessity of prevalent (intrinsic) factors, such as ‘overweight’ (high VAT), suboptimal ultra-endurance prerace performance or mal-alignment of the legs (which was only seen in one female subject suffering from a bunion). The majority of the participants was able to ‘overrun’ more or less severe overuse soft tissue syndromes in the legs and reached the finish line [2]. This indicates, that, despite the mentioned somatic parameters, other mentally based factors, such as pain resistance and personality traits, are also relevant for finishing or non-finishing a transcontinental footrace [70]. One subject (male, 61 years old) had to stop the race after stage 38 (2,601 km run) due to a high tibial stress fracture which was detected in a specific MRI on at this day (Figure 15). The astonishing thing is not the stress fracture, because this can happen to every ultra runner when starting a transcontinental race, but the fact that the major pain and massive performance (running velocity) loss had already started at stage 36. This subject ran 228 km (three stages) with a complete high tibial fracture before stopping the race, because he interpreted the pain as a soft tissue injury due to overuse and tried to ‘overrun’ it before he asked for MRI control. Another participant (female, 46 years old) showed the same behavior when running 208 km (stage 46 to 48) with a ventral pelvic ring stress fracture before diagnosis could be done with mobile MRI [2]. These examples and our prerace test on pain tolerance demonstrate that the resilience of the ultra athletes regarding pain is significantly higher than in a normal control group [70].

### Body composition and performance

In specific treadmill investigations under laboratory settings, Millet *et al*. showed that a good single ultra-marathon performance needs specific running economy depending on the ability of maximal oxygen uptake being highly correlated with citrate synthase activity and capillary network [71]. These physiological factors have not been investigated directly under race conditions in ultra-endurance events until now. Concerning this matter, only indirect parameters, such as anthropometric characteristics, are examined.

Several anthropometric factors are reported to affect performance in runners, but the presented data are inconsistent and often contradictory. Such differences are also present in the specific literature regarding anthropometrical predictors of performance outcome in ultra-marathons. Several factors are responsible for this. The numbers of volunteers are different, and in most reports they are limited and differ in gender and ethnic origin. Furthermore, the investigations are based on manifoldly different types of UM races. They can differ in the distance of running and number of stages, but also in altitude and/or external conditions.

Anthropometric parameters related to good performance are different in marathons and middle distance (half-marathon, 10 km) events [72]. Knechtle *et al*. reported that anthropometry is not associated with performance in single mono-stage UM races (24 hours [73]).

In MSUM Knechtle *et al*. found no correlation between BM or body fat (BIA) and race performance in a 17-stage MSUM (‘Deutschlandlauf 2007’, 1,200 km) [68]. In a cohort of 392 athletes, Hoffman found a significant relationship of BMI to finishing times in mono-stage UM running (161 km UM) [74]. In single marathon runners abdominal and front thigh SF are correlated [75]. The sum of eight SF-locations correlated significantly to 100 km race-time in a survey of three races in Knechtle *et al*. [76].

According to our results with a group of 22 subjects and using gold standard whole body MRI, in athletes taking part in a 64-day MSUM there are no relevant correlations between total volume, percentage fat and lean volumes of different compartments at the start and total race performance of subjects participating in TEFR09. For SAST, a significant correlation between percentage volume at the start and cumulative performance is seen at the beginning of TEFR09 (stages 1 to 8), but only at a medium to low effect size. Correlation of percentage fat and lean volumes to performance at the individual stages could only be shown in a few stages at a medium to low effect size. Looking at percentage volume distribution, the participants already started with a low percentage of body fat. Therefore, our results might confirm earlier findings of a negative relationship between the amount of subcutaneous fat tissue (thickness or volume), being the main fat tissue compartment of the body, and performance in single or multiday ultramarathon races. However, in a multistage ultramarathon over thousands of kilometers we found no relationship between body fat percentage or BM or BV and race performance using specific whole body MRI, as Knechtle *et al*. did with BIA [68]. The majority of transcontinental MSUM participants ran not for winning but for finishing the race; therefore, running velocity was a priority only for a few of them. For single UM races, the race time and, therefore, the performance plays a more important role for the ultra-athletes, and body composition and fat distribution have a more significant influence, respectively.

Similar interpretation has to be done, when looking at segmental (somatic) tissue changes in the arms, legs and trunk during TEFR09. As for adipose and lean total somatic and visceral volumes (Figure 19), we also did an analysis of the relationship between segmental tissue volume changes and race performance (results not demonstrated graphically) and detected only a small to low medium effect size for correlations between SAST of all segments (UE, TR, LE) with cumulative race performance in the first eight stages of TEFR09. So, in our investigation, all segments show a significant relationship to race performance that is similar to that of SAST over all (Figure 19) without any exceptional segment findings, which explains the inconstant finding in the literature. Knechtle *et al*. [77] found an association between triceps SF thickness and performance in female 100 km ultra-runners. Tanaka and Matsuura mentioned this for CF of the thigh in the early eighties [78].

Some ultra athletes show adaption to the intense running burden of TEFR09 with muscle (SLT) increase in the legs, although they are already specialized in ultra running. These findings were not significant in the mean. For the trunk, a mean increase of SLT could also be detected in the first third of the race. This is explained by the gluteal and psoas muscles, which are part of the active motor system of the lower extremities but anatomically are placed in the trunk in our segmentation. All lean tissue segments showed a decrease in their volumes towards the end of TEFR09, indicating the high negative energy burden of transcontinental running.

### Metabolic changes

After the first thousand kilometers the mean loss of TV per km, mainly caused by the SAST and VAT decrease, declined constantly up to more than half until the end of race (Figure 11). Despite lack of documentation of the nutrition and caloric intake but knowing that the subjects tried to ensure an optimum of energy intake, the decrease of fat volume loss can be explained by two factors: relevant metabolic changes regarding energy balancing [79] and improvement and optimization of running style during progression of the race. Not in multistage but in single stage ultra-running conditions such economical adaptations have already been shown by Millet *et al*. [80-82]. They could show significant changes of running mechanics and spring-mass behavior towards a higher mean step frequency (+4.9%) with shorter ground-feet contact time (−4.5%) and lower ground reaction force (−4.4%) due to functional leg length decrease (−13%) and increase of leg (+9.9%) and vertical stiffness (+8.6%) during the support phase of running between the early phase and the end of a 24-hour treadmill run [80]. Millet *et al*. speculated that these changes in running mechanics contributed to the overall limitation of the potentially harmful consequences of such a long-duration run on the subjects’ musculoskeletal system. Transferred to MSUM conditions, such changes in running mechanics may also contribute to the necessity of the organism to optimize the running economy to a high-end level (as low an energy consumption as possible) due to the massive negative energy burden a transcontinental race requires. The changes Millet *et al*. [80] and other researchers had measured [83,84] describe a running technique which requires only a low muscle power, because forceful eccentric load and step length are reduced. Besides the reduction of overuse risk for the musculoskeletal system this reduces the energy demand of the organism as well [85], even if the underlying mechanisms of the relation between energy cost of running and step variability remains unclear until now. If running economy could not be sacrificed in ultramarathons [86,87] and the amount of change in running mechanics depends on the duration of running and distance towards a fatigue state, respectively [81,85], it is even mandatory in transcontinental MSUM. Every subject in the TEFR-project showed a significant loss of BM and TV throughout the race, independent of the prerace overall status of body composition and performance or nutrition behavior during the race. The massive negative energy burden of a 4,500 km MSUM is also indicated by the significant loss of the grey matter in the brain [88]. The analysis of specific laboratory markers of the required blood and urine samples may give more data about the metabolic changes during TEFR09 in the near future.

### Limitations

There was no general or individual nutrition plan offered or generated for the participants of TEFR09 or subjects of the TEFR-project, respectively. The athletes had a breakfast and a dinner served in different locations at the stage destinations, but these meals were organized and oriented at the local level at the last minute. The food supply points during the stages also offered products that changed every day and the athletes took additional individual food on their own throughout the race [2]. Therefore, documentation and measurement of nutrition and caloric intake was not possible and a stringent documentation of nutrition by the subjects implied the risk of compliance problems.

Whole body mobile MRI protocols did not measure ectopic fat such as intracellular fat of organs (for example liver) and muscles (intramyocellular lipids: IMCL). For IMCL measurement, specific protocols for mobile ^1^H-MR-spectroscopy of the muscles of the lower legs were implemented in the TEFR-project [2]. However, due to the dependence of this MR-method on a stable external magnetic field around the magnetom, the analysis of mobile^1^H-MR-spectroscopy during TEFR09 did not lead to valid data and needed further development and implementation of post-imaging proof algorithms.

## Conclusions

With this mobile MRI field study a complex change in body composition during an ultra-long MSUM could be demonstrated in detail. IAAT (VAT) shows the fastest and highest decrease compared to SAST and lean tissue compartments during TEFR09. Participants lost more than half of their adipose soft tissue and even lean tissue volume decreased (mainly skeletal muscle tissue). Without exception, every subject showed a significant loss of body volume. This indicates that running an MSUM of nearly 4,500 km without any day of rest is linked with an unpreventable chronic negative energy balance due to the massive running burden. The ratio of adipose tissue contribution between the visceral and somatic compartments has a significant influence on dropping out of the race during the first third in a MUSM due to overuse injuries of the myotendinous fascial system of the legs. Body volume or body mass and, therefore, fat volume has no correlation with the performance of ultra-athletes finishing a 64-stage UM. Two- and three-compartment methods, such as bioelectrical impedance analysers and skinfold-equations, cannot give estimations about the relationship between the visceral and somatic compartments and, therefore, cannot measure the most sensitive anthropometric predictor of not finishing a MSUM: VAT. Running economy is mandatory for transcontinental MSUM races and, even in well trained ultra-athletes, such events lead to further adaptation of running mechanics and to metabolic changes as performance analysis compared to body composition changes throughout the race indicates.

## Abbreviations

ABM: Adipose bone marrow; ANOVA: Analysis of variance; BIA: Bioelectrical impedance analysis; BM: Body mass; BMI: Body mass index; CF: Body circumference; CF: Body circumference; CHESS: Chemical shift selective (imaging); CCP: Pearson correlation coefficient; CCS: Spearman-rho correlation coefficient; DEXA: Dual-energy X-ray absorptiometry; F: Finisher; IAAT: Intraabdominal adipose tissue: retroperitoneal and intraperitoneal fat depots; IMAT: Intermuscular adipose tissue; IMCL: Intramyocellular lipids; INF: Intraluminal nutrition fat in the gastrointestinal tract; LT-LE: Lean tissue volume of lower extremities; LT-TR: Lean soft tissue volume of trunk; LT-UE: Lean soft tissue volume of upper extremities; MAT: Intrathoracic, mainly mediastinal adipose tissue; max: Maximum; MI: Measurement interval; min: Minimum; MR: Magnetic resonance; MRI: Magnetic resonance imaging; MSUM: Multistage ultramarathon; NF: Non-finisher; PRY: Prerace years of regular endurance running; PRR: Prerace records; PRRM: Prerace record in marathon; PRR50km: Prerace record in 50 km-races; PRR100km: Prerace record in 100 km-races; PRR6hr: Prerace record in 6 hours-races; PRR12hr: Prerace record in 12 hours-races; PRR24hr: Prerace record in 24 hours-races; PRT: Prerace training; PRTInt08: Prerace training intensity (km per week) in 2008; PRTVol08: Prerace training volume (hours per week) in 2008; PRTVol09: Prerace training volume (hours per week) in 2009; PRTTime08: Prerace training hours per week in 2008; PRTTime09: Prerace training hours per week in 2009; SAST: Somatic adipose soft tissue; SAT: Somatic adipose tissue; SCAT: Subcutaneous adipose tissue; SD: Standard deviation; SF: Skinfold thickness; SLT: Somatic lean tissue: mostly muscles; TAST: Total adipose soft tissue; TAT: Total adipose tissue; TEFR09: Transeurope Footrace 2009; TEFR-project: Transeurope Footrace Project; TLT: Total lean tissue; TSV: Total somatic volume; TV: Total volume of the body; TV-LE: Total volume of lower extremities; TV-TR: Total volume of trunk; TV-UE: Total volume of upper extremities; TVV: Total visceral volume; UM: Ultramarathon; VAT: Visceral adipose tissue; VLT: Visceral lean tissue: includes lean tissue of intrathoracic and intraabdominal organs.

## Competing interests

The authors declare they have no competing interests.

## Authors’ contributions

All authors of this manuscript made substantial contributions to the conception and design or acquisition, analysis and interpretation of data; all revised it critically for important intellectual content and gave final approval to the version to be published. SUHW conceived the study, implemented the project, participated in the data collection (MRI measurements), data evaluation with statistical analysis and drafted the manuscript. BC participated in the implementation of the project and in the data collection (MRI measurements). KK participated in the data evaluation. WC participated in the data evaluation. WH participated mainly in the data collection (MRI measurements) and in the implementation of the project. BHJ participated in the implementation of the project. MJ participated in the design of the study, its technical implementation and data evaluation. All authors read and approved the final manuscript.

## Pre-publication history

The pre-publication history for this paper can be accessed here:

http://www.biomedcentral.com/1741-7015/11/122/prepub

## References

[B1] Knechtle B Enggist A Jehle T Energy turnover at the Race across America (RAAM): a case report Int J Sports Med 2005 26 499 503 10.1055/s-2004-821136 16037895

[B2] Schütz UH Schmidt-Trucksäss A Knechtle B Machann J Ehrhardt M Wiedelbach H Freund W Gröninger S Brunner H Schulze I Brambs HJ Billich C The Transeurope Footrace Project: Longitudinal data acquisition in a cluster randomized mobile MRI observational cohort study on 44 endurance runners at a 64-stage 4,486km transcontinental ultramarathon BMC Med 2012 19 78 22812450 10.1186/1741-7015-10-78 PMC3409063

[B3] Transeurope Footracehttp://www.transeurope-footrace.org

[B4] Schulze I TransEurope-FootRace 2009: Bari - Nordkap - 4.487,7 km in 64 Tagesetappen 2010 1 Leipzig, Germany: Engelsdorfer Verlag

[B5] Brodie DA Stewart AD Body composition measurement: a hierarchy of methods J Pediatr Endocrinol Metab 1999 12 801 816 10614537 10.1515/jpem.1999.12.6.801

[B6] Lee RC Wang Z Heo M Ross R Janssen I Heymsfield SB Total-body skeletal muscle mass: development and cross-validation of anthropometric prediction models Am J Clin Nutr 2000 72 796 803 10966902 10.1093/ajcn/72.3.796

[B7] Janssen I Heymsfield SB Baumgartner RN Ross R Estimation of skeletal muscle mass by bioelectrical impedance analysis J Appl Physiol 2000 89 465 471 10926627 10.1152/jappl.2000.89.2.465

[B8] Chan DC Watts GF Barrett PHR Burke V Waist circumference, waist-to-hip ratio and body mass index as predictors of adipose tissue compartments in men QJM 2003 96 441 447 10.1093/qjmed/hcg069 12788963

[B9] Gualdi-Russo E Toselli S Influence of various factors on the measurement of multifrequency bioimpedance Homo 2002 53 1 16 10.1078/0018-442X-00035 12365353

[B10] Lee SY Gallagher D Assessment methods in human body composition Curr Opin Clin Nutr Metab Care 2008 11 566 572 10.1097/MCO.0b013e32830b5f23 18685451 PMC2741386

[B11] Daniel JA Sizer PS Jr Latman NS Evaluation of body composition methods for accuracy Biomed Instrum Technol 2005 39 397 405 10.2345/0899-8205(2005)39[397:EOBCMF]2.0.CO;2 16248452

[B12] Vasudev S Mohan A Mohan D Farooq S Raj D Mohan V Validation of body fat measurement by skin folds and two bioelectric impedance methods with DEXA – the Chennai Urban Rural Epidemiology Study (CURES-3) J Assoc Physicians India 2004 52 877 881 15906838

[B13] Broeder CE Burrhus KA Svanevik LS Volpe J Wilmore JH Assessing body composition before and after resistance or endurance training Med Sci Sports Exerc 1997 29 705 712 10.1097/00005768-199705000-00019 9140911

[B14] Vogt FM Ruehm S Hunold P de Greiff A Nuefer M Barkhausen J Ladd SC Rapid total body fat measurement by magnetic resonance imaging: quantification and topography Rofo 2007 179 480 486 10.1055/s-2007-962833 17377875

[B15] Ross R Léger L Morris D de Guise J Guardo R Quantification of adipose tissue by MRI: relationship with anthropometric variables J Appl Physiol 1992 72 787 795 1559959 10.1152/jappl.1992.72.2.787

[B16] Thomas EL Saeed N Hajnal JV Brynes A Goldstone AP Frost G Bell JD Magnetic resonance imaging of total body fat J Appl Physiol 1998 85 1778 1785 9804581 10.1152/jappl.1998.85.5.1778

[B17] Ludescher B Machann J Eschweiler GW Vanhöfen S Maenz C Thamer C Claussen CD Schick F Correlation of fat distribution in whole body MRI with generally used anthropometric data Invest Radiol 2009 44 712 719 10.1097/RLI.0b013e3181afbb1e 19809346

[B18] Abate N Burns D Peshock RM Garg A Grundy SM Estimation of adipose tissue mass by magnetic resonance imaging: validation against dissection in human cadavers J Lipid Res 1994 35 1490 1496 7989873

[B19] Ross R Shaw KD Martel Y de Guise J Avruch L Adipose tissue distribution measured by magnetic resonance imaging in obese women Am J Clin Nutr 1993 57 470 475 8460599 10.1093/ajcn/57.4.470

[B20] Poon CS Szumowski J Plewes DB Ashby P Henkelman RM Fat/water quantitation and differential relaxation time measurement using chemical shift imaging technique Magn Reson Imaging 1989 7 369 382 10.1016/0730-725X(89)90486-4 2811618

[B21] Lunati E Marzola P Nicolato E Sbarbati A In-vivo quantitative hydrolipidic map of perirenal adipose tissue by chemical shift imaging at 4.7 Tesla Int J Obes Relat Metab Disord 2001 25 457 461 10.1038/sj.ijo.0801262 11319646

[B22] Schick F Machann J Brechtel K Strempfer A Klumpp B Stein DT Jacob S MRI of muscular fat Magn Reson Med 2002 47 720 727 10.1002/mrm.10107 11948733

[B23] Huang TY Chung HW Wang FN Ko CW Chen CY Fat and water separation in balanced steady-state free precession using the Dixon method Magn Reson Med 2004 51 243 247 10.1002/mrm.10686 14755647

[B24] Donnelly LF O’Brien KJ Dardzinski BJ Poe SA Bean JA Holland SK Daniels SR Using a phantom to compare MR techniques for determining the ratio of intraabdominal to subcutaneous adipose tissue AJR Am J Roentgenol 2003 180 993 998 10.2214/ajr.180.4.1800993 12646443

[B25] Machann J Thamer C Schnoedt B Haap M Haring HU Claussen CD Stumvoll M Fritsche A Schick F Standardized assessment of whole body adipose tissue topography by MRI J Magn Reson Imaging 2005 21 455 462 10.1002/jmri.20292 15778954

[B26] Würslin C Machann J Rempp H Claussen C Yang B Schick F Topography mapping of whole body adipose tissue using a fully automated and standardized procedure J Magn Reson Imaging 2010 31 430 439 10.1002/jmri.22036 20099357

[B27] Stephens MA Test of fit for the logistic distribution based on the empirical distribution function Biometrika 1979 66 591 595 10.1093/biomet/66.3.591

[B28] D’Agostino RB Belanger A D’Agostino RB Jr A suggestion for using powerful and informative test of normality Am Stat 1990 44 316 321

[B29] Yazici B Yolacan S A comparison of various tests of normality J Stat Comput Simul 2007 77 175 183 10.1080/10629360600678310

[B30] Cohen J A power primer Psychol Bull 1992 112 155 159 19565683 10.1037//0033-2909.112.1.155

[B31] Boettcher M Machann J Stefan N Thamer C Häring HU Claussen CD Fritsche A Schick F Intermuscular adipose tissue (IMAT): association with other adipose tissue compartments and insulin sensitivity J Magn Reson Imaging 2009 29 1340 1345 10.1002/jmri.21754 19422021

[B32] Gray DS Fujioka K Colletti PM Kim H Devine W Cuyegkeng T Pappas T Magnetic-resonance imaging used for determining fat distribution in obesity and diabetes Am J Clin Nutr 1991 54 623 627 1897468 10.1093/ajcn/54.4.623

[B33] Bale P Rowell S Colley E Anthropometric and training characteristics of female marathon runners as determinants of distance running performance J Sports Sci 1985 3 115 126 10.1080/02640418508729741 4094022

[B34] Raschka C Plath M Body fat compartment and its relationship to food intake and clinical chemical parameters during extreme endurance performance Schweiz Z Sportmed 1992 40 13 25 1561538

[B35] Reynolds RD Lickteig JA Deuster PA Howard MP Conway JM Pietersma A De Stoppelaar J Deurenberg P Energy metabolism increases and regional body fat decreases while regional muscle mass is spared in humans climbing Mt Everest. J Nutr 1999 129 1307 1314 10.1093/jn/129.7.1307 10395591

[B36] Helge JW Lundby C Christensen DL Langfort J Messonnier L Zacho M Andersen JL Saltin B Skiing across the Greenland icecap: divergent effects on limb muscle adaptations and substrate oxidation J Exp Biol 2003 206 1075 1083 10.1242/jeb.00218 12582149

[B37] Frykman PN Harman EA Opstad PK Hoyt RW DeLany JP Friedl KE Effects of a 3-month endurance event on physical performance and body composition: the G2 trans-Greenland expedition Wilderness Environ Med 2003 14 240 248 10.1580/1080-6032(2003)14[240:EOAMEE]2.0.CO;2 14719859

[B38] Höchli D Schneiter T Ferretti G Howald H Claassen H Moia C Atchou G Belleri M Veicsteinas A Hoppeler H Loss of muscle oxidative capacity after an extreme endurance run: the Paris-Dakar Foot-Race Int J Sports Med 1995 16 343 346 10.1055/s-2007-973017 7591382

[B39] Knechtle B Knechtle P Andonie JL Kohler G Influence of anthropometry on race performance in extreme endurance triathletes: World Challenge Deca Iron Triathlon 2006 Br J Sports Med 2007 41 644 648 10.1136/bjsm.2006.035014 17556527 PMC2465179

[B40] Bircher S Enggist A Jehle T Effects of an extreme endurance race on energy balance and body composition: a case study J Sports Sci Med 2006 5 154 162 24198693 PMC3818668

[B41] Lehmann M Huonker M Dimeo F Serum amino acid concentrations in nine athletes before and after the 1993 Colmar Ultra Triathlon Int J Sports Med 1995 16 155 159 10.1055/s-2007-972984 7649705

[B42] Knechtle B Bircher S Changes in body composition during an extreme endurance run Praxis 2005 94 371 377 10.1024/0369-8394.94.10.371 15794360

[B43] Dressendorfer RH Wade CE Effects of a 15-d race on plasma steroid levels and leg muscle fitness in runners Med Sci Sports Exerc 1991 23 954 958 1956271

[B44] Nagel D Seiler D Franz H Leitzmann C Jung K Effects of an ultra-long-distance (1000 km) race on lipid metabolism Eur J Appl Physiol 1989 59 16 20 10.1007/BF02396574 2583145

[B45] Väänänen II Vihko V Physiological and psychological responses to 100 km crosscountry skiing during 2 days J Sports Med Phys Fitness 2005 45 301 305 16230981

[B46] Raschka C Plath M Cerull R Bernhard W Jung K Leitzmann C The body muscle compartment and its relationship to food absorption and blood chemistry during an extreme endurance performance Z Ernährungswiss 1991 30 276 288 1788995 10.1007/BF01651957

[B47] Knechtle B Salas OF Andonie JL Kohler G Effect of a multistage ultra-endurance triathlon on body composition: World Challenge Deca Iron Triathlon 2006 Br J Sports Med 2008 42 121 125 17601765 10.1136/bjsm.2007.038034

[B48] Knechtle B Duff B Schule I Kohler G A multi-stage ultra-endurance run over 1,200 km leads to a continuous accumulations of total body water J Sports Sci Med 2008 7 357 364 24149903 PMC3761892

[B49] Martin AD Daniel MZ Drinkwater DT Clarys JP Adipose tissue density, estimated adipose lipid fraction and whole body adiposity in male cadavers Int J Obes Relat Metab Disord 1994 18 79 83 8148928

[B50] Mernagh JR Harrison JE Krondl A McNeill KG Shepard RJ Composition of lean tissue in healthy volunteers for nutritional studies in health and disease Nutrition Res 1986 6 499 507 10.1016/S0271-5317(86)80103-8

[B51] Wells JC Williams JE Chomtho S Darch T Grijalva-Eternod C Kennedy K Haroun D Wilson C Cole TJ Fewtrell MS Pediatric reference data for lean tissue properties: density and hydration from age 5 to 20 y Am J Clin Nutr 2010 91 610 618 10.3945/ajcn.2009.28428 20089731

[B52] Schutte JE Townsend EJ Hugg J Shoup RF Malina RM Blomqvist CG Density of lean body mass is greater in blacks than in whites J Appl Physiol 1984 56 1647 1649 10.1063/1.334152 6735823

[B53] Enzi G Digito M Marin R Carraro R Baritussio A Manzato E Mediastino-abdominal lipomatosis: deep accumulation of fat mimicking a respiratory disease and ascites. Clinical aspects and metabolic studies in vitro Q J Med 1984 53 453 463 6515001

[B54] Brochu M Starling RD Tchernof A Matthews DE Garcia-Rubi E Poehlman ET Visceral adipose tissue is an independent correlate of glucose disposal in older obese postmenopausal women J Clin Endocrinol Metab 2000 85 2378 2384 10.1210/jc.85.7.2378 10902782

[B55] Kahn BB Flier JS Obesity and insulin resistance J Clin Invest 2000 106 473 481 10.1172/JCI10842 10953022 PMC380258

[B56] Wajchenberg BL Subcutaneous and visceral adipose tissue: their relation to the metabolic syndrome Endocr Rev 2000 21 697 738 10.1210/er.21.6.697 11133069

[B57] Sironi AM Gastaldelli A Mari A Ciociaro D Postano V Buzzigoli E Ghione S Turchi S Lomabardi M Ferrannini E Visceral fat in hypertension: influence on insulin resistance and β-cell function Hypertension 2004 44 127 133 10.1161/01.HYP.0000137982.10191.0a 15262911

[B58] Yusuf S Hawken S Ounpuu S Dans T Avezum A Lanas F McQueen M Budaj A Pais P Varigos J Lisheng L INTERHEART Study Investigators Effect of potentially modifiable risk factors associated with myocardial infarction in 52 countries (the INTERHEART study): case–control study Lancet 2004 364 937 952 10.1016/S0140-6736(04)17018-9 15364185

[B59] Montague CT O’Rahilly S The perils of portliness: causes and consequences of visceral adiposity Diabetes 2000 49 883 888 10.2337/diabetes.49.6.883 10866038

[B60] Kern PA Ranganathan S Li C Wood L Ranganathan G Adipose tissue tumor necrosis factor and interleukin-6 expression in human obesity and insulin resistance Am J Physiol Endocrinol Metab 2001 280 E745 E751 11287357 10.1152/ajpendo.2001.280.5.E745

[B61] Marette A Molecular mechanisms of inflammation in obesity-linked insulin resistance Int J Obes Relat Metab Disord 2003 27 S46 S48 14704744 10.1038/sj.ijo.0802500

[B62] Sharma AM Mediastinal fat, insulin resistance, and hypertension Hypertension 2004 44 117 118 10.1161/01.HYP.0000137993.70745.82 15249549

[B63] Lee WJ Fattal G Mediastinal lipomatosis in simple obesity Chest 1976 70 308 309 10.1378/chest.70.2.308 947702

[B64] Price JE Jr Rigler LG Widening of the mediastinum resulting from fat accumulation Radiology 1970 96 497 500 4917286 10.1148/96.3.497

[B65] Stummvoll HK Wolf A Pinggera WF Lobenwein E Seidl G Rare localizations of fat deposition in iatrogenous Cushing’s syndrome Munch Med Wochenschr 1976 118 445 446 817178

[B66] Kopelman PG The effects of weight loss treatments on upper and lower body fat Int J Obes Relat Metab Disord 1997 21 619 625 10.1038/sj.ijo.0800458 15481759

[B67] Martin AD Janssens V Caboor D Clarys JP Marfell-Jones MJ Relationships between visceral, trunk and whole-body adipose tissue weights by cadaver dissection Ann Hum Biol 2003 30 668 677 10.1080/03014460310001599590 14675908

[B68] Knechtle B Duff B Schulze I Rosemann T Senn O Anthropometry and pre-race experience of finishers and nonfinishers in a multistage ultra-endurance run–Deutschlandlauf 2007 Percept Mot Skills 2009 109 105 118 10.2466/pms.109.1.105-118 19831091

[B69] Freund W Weber F Billich C Schütz UH The foot in multistage ultra marathon runners: Experience in a cohort study of 22 participants of the Trans Europe Footrace project with mobile MRI BMJ Open 2012 2 10.1136/bmjopen-2012-001118 PMC3364457 22619270

[B70] Freund W Weber F Billich C Birklein F Breimhorst M Schütz UH Ultra marathon runners are different. Investigations into pain tolerance and personality traits of participants of the TransEurope FootRace 2009 Pain Pract 2013 10.1111/papr.12039 23368760

[B71] Millet GY Banfi JC Kerherve H Morin JB Vincent L Estrade C Geyssant A Feasson L Physiological and biological factors associated with a 24 h treadmill ultra-marathon performance Scand J Med Sci Sports 2011 21 54 61 10.1111/j.1600-0838.2009.01001.x 19883385

[B72] Maldonado S Mujika I Padilla S Influence of body mass and height on the energy cost of running in highly trained middle- and long-distance runners Int J Sports Med 2002 23 268 272 10.1055/s-2002-29083 12015627

[B73] Knechtle B Wirth A Knechtle P Zimmermann K Kohler G Personal best marathon performance is associated with performance in a 24-h run and not anthropometry or training volume Br J Sports Med 2009 43 836 839 10.1136/bjsm.2007.045716 18385194

[B74] Hoffman MD Anthropometric characteristics of ultramarathoners Int J Sports Med 2008 29 808 811 10.1055/s-2008-1038434 18401807

[B75] Arrese AL Ostáriz ES Skinfold thicknesses associated with distance running performance in highly trained runners J Sports Sci 2006 24 69 76 10.1080/02640410500127751 16368615

[B76] Knechtle B Knechtle P Rosemann T Senn O What is associated with race performance in male 100-km ultra-marathoners - anthropometry, training or marathon best time? J Sports Sci 2011 23 1 7 10.1080/02640414.2010.541272 21360403

[B77] Knechtle B Knechtle P Rosemann T Lepers R Predictor variables for a 100 km race time in female ultra-marathoners Medicina Sportiva 2010 14 214 220 10.2478/v10036-010-0035-0 21319608

[B78] Tanaka K Matsuura Y A multivariate analysis of the role of certain anthropometric and physiological attributes in distance running Ann Hum Biol 1982 9 473 482 10.1080/03014468200006001 7137943

[B79] Tomaszewski M Charchar FJ Przybycin M Crawford L Wallace AM Gosek K Lowe GD Zukowska-Szczechowska E Grzeszczak W Sattar N Dominiczak AF Strikingly low circulating CRP concentrations in ultramarathon runners independent of markers of adiposity: how low can you go? Arterioscler Thromb Vasc Biol 2003 23 1640 1644 10.1161/01.ATV.0000087036.75849.0B 12869354

[B80] Morin JB Samozino P Millet GY Changes in running kinematics, kinetics, and spring-mass behavior over a 24-h run Med Sci Sports Exerc 2011 43 829 836 20962690 10.1249/MSS.0b013e3181fec518

[B81] Degache F Guex K Fourchet F Morin JB Millet GP Tomazin K Millet GY Changes in running mechanics and spring-mass behaviour induced by a 5-hour hilly running bout J Sports Sci 2013 31 299 304 10.1080/02640414.2012.729136 23051041

[B82] Borrani F Candau R Perrey S Millet GY Millet GP Rouillon JD Does the mechanical work in running change during the VO2 slow component? Med Sci Sports Exerc 2003 35 50 57 10.1097/00005768-200301000-00009 12544635

[B83] Brisswalter J Legros P Durand M Running economy, preferred step length correlated to body dimensions in elite middle distance runners J Sports Med Phys Fitness 1996 36 7 15 8699842

[B84] Svedenhag J Sjödin B Body-mass-modified running economy and step length in elite male middle- and long-distance runners Int J Sports Med 1994 15 305 310 10.1055/s-2007-1021065 7822068

[B85] Candau R Belli A Millet GY Georges D Barbier B Rouillon JD Energy cost and running mechanics during a treadmill run to voluntary exhaustion in humans Eur J Appl Physiol Occup Physiol 1998 77 479 485 10.1007/s004210050363 9650730

[B86] Millet GP Economy is not sacrificed in ultramarathon runners J Appl Physiol 2012 113 686 10.1152/japplphysiol.00642.2012 22896682

[B87] Millet GY Hoffman MD Morin JB Sacrificing economy to improve running performance–a reality in the ultramarathon? J Appl Physio 2012 113 507 509 10.1152/japplphysiol.00016.2012 22492933

[B88] Freund W Faust S Birklein F Gaser C Wunderlich AP Mueller M Billich C Juchems MS Schmitz BL Groen G Schütz UH Substantial and reversible brain gray matter reduction but no acute brain lesions in ultramarathon runners: experience from the TransEurope-FootRace Project BMC Med 2012 21 10 170 10.1186/1741-7015-10-170 PMC3566943 23259507

